# Designing Efficient Inverted Perovskite Solar Cells with Self-Assembled Monolayer Hole Transport Layers

**DOI:** 10.1007/s40820-026-02327-0

**Published:** 2026-08-10

**Authors:** Xinyuan Feng, Qiuying Su, Long Zhou, Jiaojiao Zhang, Dazheng Chen, Weidong Zhu, He Xi, Chunfu Zhang, Jincheng Zhang, Yue Hao

**Affiliations:** State Key Laboratory of Wide-Bandgap Semiconductor Devices and Integrated Technology, Xidian University, Xi’an, 710071 People’s Republic of China

**Keywords:** Self-assembled monolayer, Perovskite solar cell, Hole transport layer, Interfacial engineering

## Abstract

Self-assembled monolayers (SAMs) have emerged as highly versatile interfacial materials in perovskite solar cells (PSCs), offering tunable molecular structures, favorable energy-level alignment, high optical transparency, and minimized non-radiative recombination losses. With the rapid advancement of inverted (p-i-n) PSC architectures, SAM-based hole-selective contacts have demonstrated distinct advantages in achieving superior power conversion efficiency, cost-effective fabrication, and strong compatibility with scalable manufacturing processes. This review first summarizes the evolution of SAM applications, with a particular focus on SAM-based materials in p-i-n PSCs. Subsequently, the fundamental aspects of SAMs are systematically discussed. Further, common preparation methods of SAMs are reviewed, along with the key challenges encountered in achieving uniform SAM coating. Based on this, recent progress in SAM-based PSCs is comprehensively summarized, including their applications in high-efficiency single-junction devices, perovskite tandem solar cells, and large-area photovoltaic modules. The crucial roles of SAMs in energy-level modulation, interfacial modification, defect passivation, and charge transport are highlighted. Finally, the remaining challenges and future prospects of SAMs in inverted PSCs are discussed, with particular emphasis on interfacial stability and long-term operational reliability. This review aims at providing systematic insights and guidance for the further development of SAM-based inverted PSCs.

## Highlights

- This review summarizes the development of self-assembled monolayers (SAMs), with a special focus on their applications in inverted (p-i-n) perovskite solar cells.
- This review discusses the structure, preparation methods, interfacial roles of SAMs, as well as the common problems encountered during their fabrication process.
- This review highlights recent advances of SAM-based inverted perovskite solar cells in efficient single-junction, tandem, and large-area modules and outlines key challenges and future prospects of SAM applications.

## Graphical abstract

**Figure:**
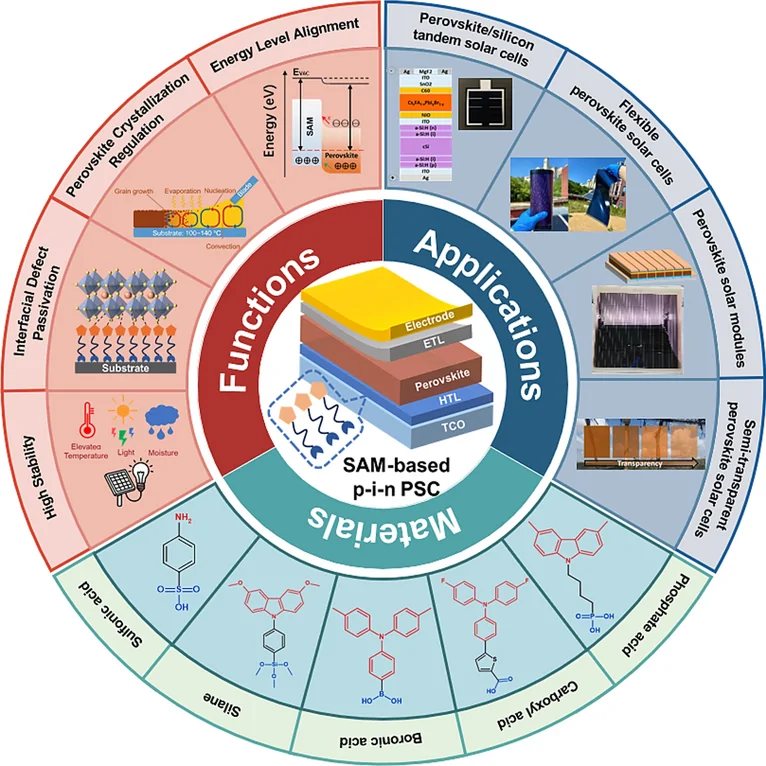

## Introduction

The relentless increase in global demand for fossil fuels, coupled with their finite availability and detrimental environmental consequences, has intensified the pursuit of sustainable green-energy alternatives. Solar energy, an essentially inexhaustible resource, has progressively emerged as a research focus. At present, silicon-based solar cells dominate the photovoltaic market; however, they encounter intrinsic challenges such as energy-intensive fabrication processes, environmental contamination, and stagnating efficiency breakthroughs. In recent decades, scientists have endeavored to identify innovative materials for advancing high-efficiency photoelectric conversion. In 2009, Miyasaka and his colleagues [1] pioneered solar cells using MAPbI_3_ as the active light absorbing layer, achieving a power conversion efficiency (PCE) of 3.8%. Since then, perovskites have rapidly emerged as a cornerstone of solar cell research, driven by their exceptional optoelectronic properties and transformative potential in photovoltaic technology. These properties include tunable bandgaps, high absorption coefficients, low exciton binding energies, long charge diffusion lengths, and prolonged carrier lifetimes. Furthermore, perovskite thin films can be readily fabricated through low-temperature solution-based processes. Over the past decade, the efficiency of perovskite solar cells (PSCs) has escalated swiftly, surpassing 27% by 2025, approaching the theoretical efficiency limit of 29.4% for silicon-based solar cells [2, 3].

**Fig. 1:**
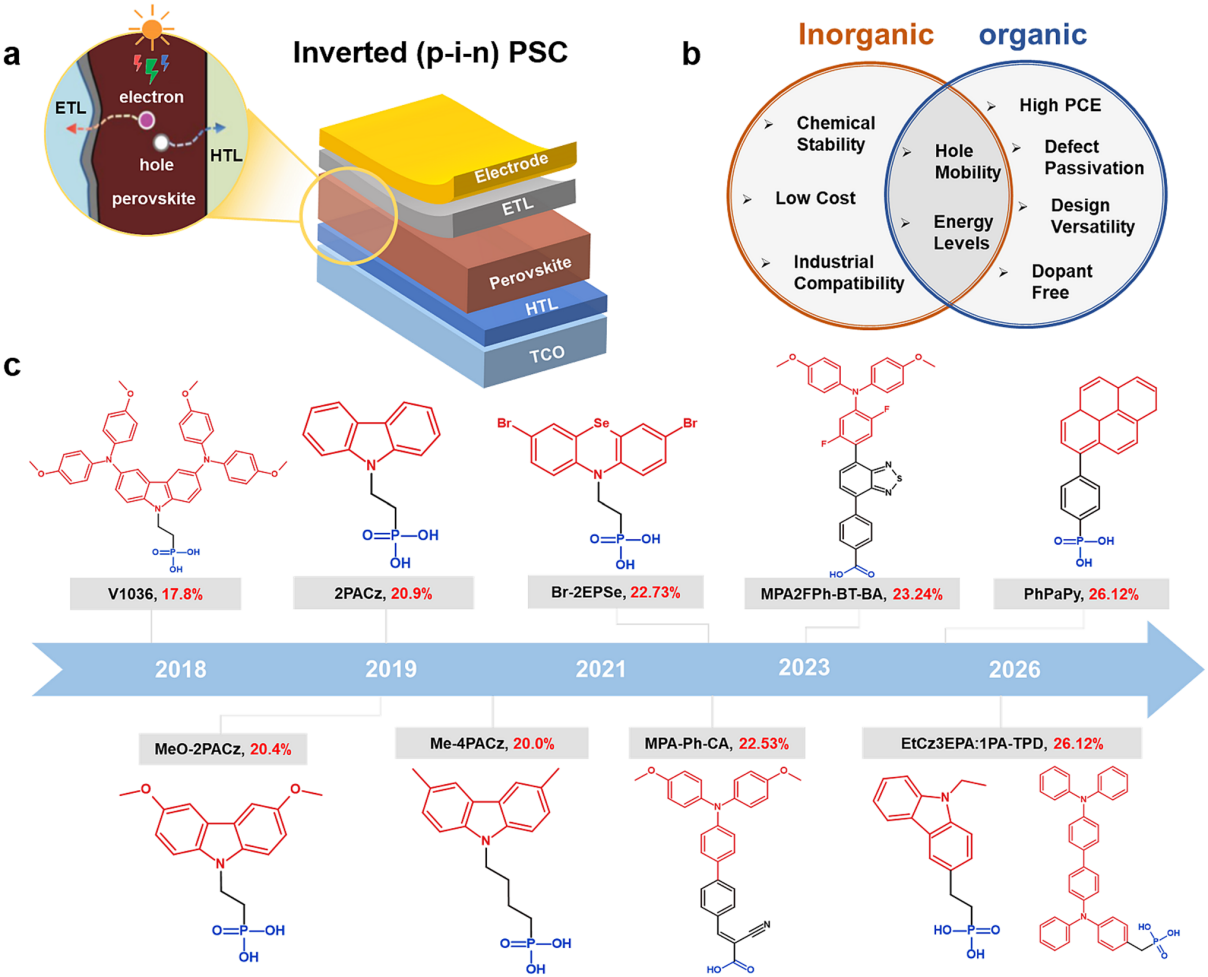
**a** Framework structures of inverted PSCs and their working principle. **b** Properties which a practicable for inorganic and organic HTMs. **c** Main progress of efficient SAM-based-HTLs in PSCs and their chemical structures, including V1036 [8], MeO-2PACz [9], 2PACz [9], Me-4PACz [10], MPA-Ph-CA [11], Br-2EPSe [12], MPA2FPh-BT-BA [13], PhPaPy [14], EtCz3EPA:1PA-TPD [15]

A typical PSC comprises an electron transport layer (ETL), a hole transport layer (HTL), and a perovskite photoactive layer sandwiched between them. In such devices, when the ETL is deposited onto a transparent conducting oxide (TCO) substrate, typically tin-doped indium oxide (ITO) or fluorine-doped tin oxide (FTO), and the HTL interfaces with the metal electrode, the configuration is termed an n-i-p structure. Conversely, the inverted p-i-n configuration places the HTL adjacent to the TCO, while the ETL interfaces directly with the metal electrode, as illustrated in Fig. 1a. In inverted PSCs, incident light first passes through the transparent electrode and is absorbed by the perovskite layer, generating electron–hole pairs. The holes are efficiently extracted by the HTL and collected at the anode, while the electrons are transferred through the ETL toward the cathode. The built-in electric field between the two electrodes drives charge separation and transport, thereby converting solar energy into electrical energy. Hole transport materials (HTMs) can be strategically engineered to both passivate defects within perovskite layers and provide protective barriers against environmental degradation in n-i-p device architectures. In p-i-n architectures, HTMs assume an even more pivotal role, serving as substrates for perovskite deposition and directly regulating thin-film growth and crystallization kinetics. Consequently, optimizing the selection of HTMs alongside precise engineering of the HTL/perovskite interface is crucial during device fabrication, as both factors work synergistically to minimize resistive losses and enhance hole extraction efficiency. Conventional HTMs fall into two primary categories: inorganic compounds and organic materials, with the latter further subdivided into polymers and small molecules. Figure 1b illustrates that both organic and inorganic HTMs demonstrate a range of distinct advantages and limitations, and Fig. 1c displays the evolutionary trajectory of dominant HTMs in PSCs. Concerns over the high cost and limited stability of organic HTMs initially hindered their adoption, prompting a shift toward more robust inorganic alternatives. However, despite the demonstrated robustness of inorganic HTMs, they continue to underperform compared to their organic counterparts in terms of achieving high PCEs. This advancement spurred the development of polymeric HTMs, which integrate enhanced charge-transport capability with improved long-term stability. Self-assembled monolayers (SAMs) have recently become a research hotspot, due to their multifunctional advantages. Compared with conventional HTLs such as poly(3,4-ethylenedioxythiophene) polystyrene sulfonate (PEDOT:PSS), poly(triarylamine) (PTAA), and nickel oxide (NiO_*x*_), SAMs exhibit several unique advantages in inverted PSCs. PEDOT:PSS, with its pronounced acidity and hygroscopicity, can corrode ITO and accelerate device degradation, whereas SAMs provide superior energy-level alignment without adversely affecting the substrate environment [4]. PTAA typically exhibits hydrophobic behavior that limits perovskite coverage and compromises film quality, while SAMs can optimize perovskite crystallization and effectively passivate buried interfacial defects [5]. Moreover, the conductivity of NiO_*x*_ thin films is relatively poor, and redox reactions occur at the NiO_*x*_/perovskite interface. By contrast, the strong chemical interaction between SAMs and oxide substrates ensures effective anchoring, thereby regulating the substrate work function (WF) and improving interfacial charge transport [6]. The intrinsic dipole moment of SAM molecules can induce a shift in WF of the substrate, thereby facilitating more efficient charge extraction and injection. Furthermore, the heteroatoms (S or O) and functional substituents (such as amine groups or halogens) present in the terminal groups of SAM molecules can effectively modulate the nucleation of perovskite precursor species and guide the subsequent crystal growth process. Such interactions foster improved crystallinity, reduce defect density, suppress non-radiative recombination, and enhance interfacial charge-transfer dynamics [7].

This review provides a systematic examination of the classification of SAMs, their exceptional performance as HTLs in PSCs, and foreseeable future challenges. Currently, SAMs are primarily categorized based on their anchoring groups. Furthermore, SAMs can be easily customized through strategies such as terminal functional group modification or integration with multiple HTM composites, enabling precise adaptation to perovskites with tunable bandgaps and diverse device architectures. Such molecular engineering enhances PCE, suppresses phase segregation, optimizes energy-level alignment, and improves uniformity of perovskite film during large-area fabrication. Finally, we present our perspectives on critical challenges for advancing SAM-based HTL technologies.

## Self-Assembled Monolayers

### Structure of SAMs

**Fig. 2:**
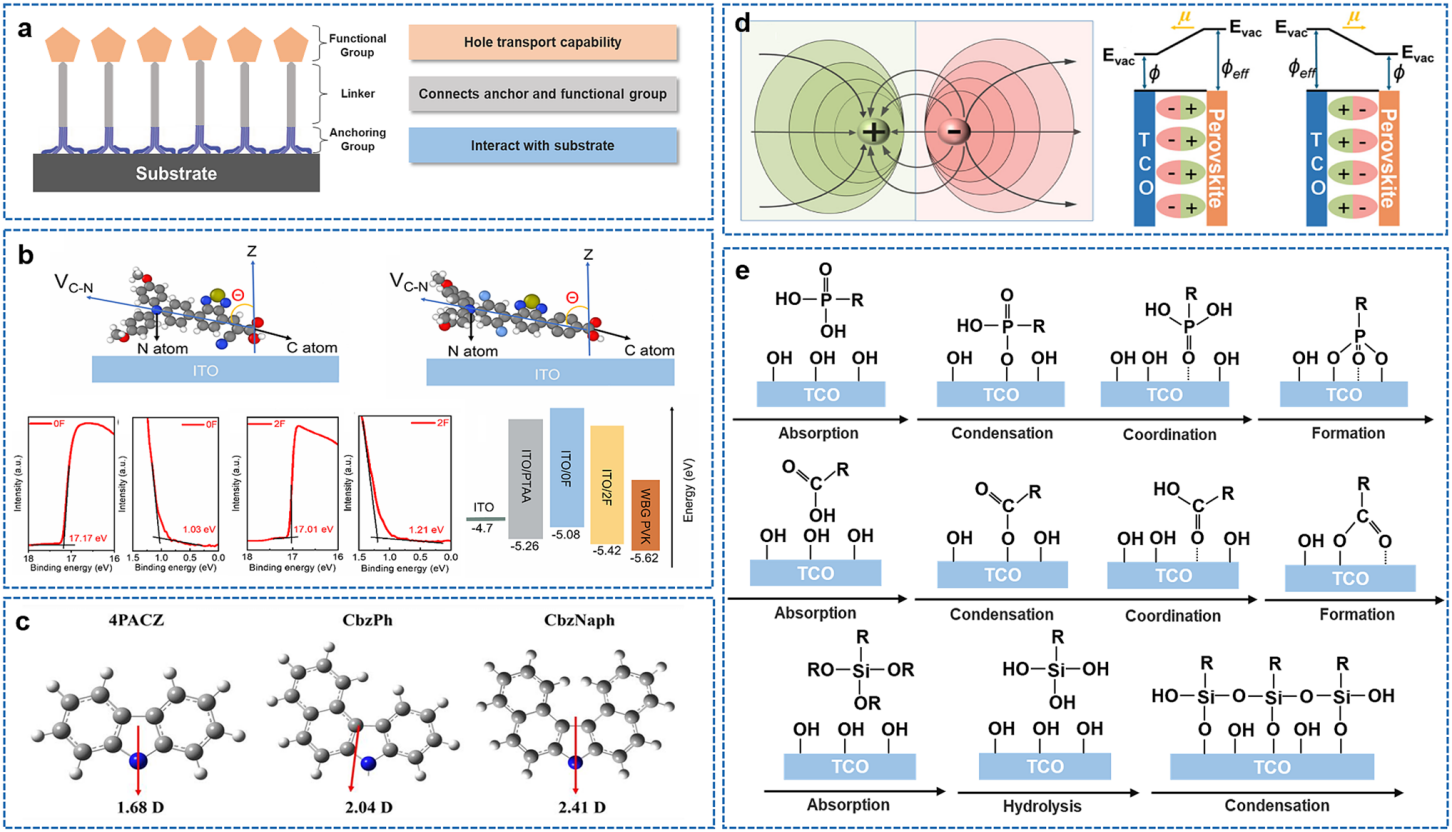
**a** Schematic diagram of SAM structure. **b** Molecular dynamics simulation of two SAMs on ITO substrate (top). UPS results and energetic diagram of HTLs deposited on ITO and WBG PVK (bottom) [13]. Copyright 2023 The Author(s), under exclusive license to Springer Nature Limited. **c** Calculated molecular structures and dipoles of SAMs [18]. Copyright 2022 Wiley–VCH. **d** Depiction of a dipole and its effects on band bending (left) and Schematic diagram of the SAM mechanism (right) [19]. Copyright 2024 The Royal Society of Chemistry. **e** Adhesion mechanism of phosphonic acid (top), carboxylic acid (middle), and silane-based SAMs with different binding modes (bottom)

The defining characteristics of SAMs stem from the chemical functionalization of their constituent molecules or ligands, which endows them with a higher affinity for the substrate surface. Consequently, one or several layers of organic molecules spontaneously anchor onto the surface of a solid substrate, a process primarily governed by the molecular composition of the self-assembled film and the chemical properties of the substrate. Driven by the thermodynamically favorable self-assembly process, molecules in self-assembled films preferentially align vertically on the substrate surface, yielding a highly ordered SAM structure. Based on their packing density and organizational order, SAMs can be classified into crystalline, semicrystalline, or amorphous structures. Compared with conventional p-type semiconductor hole-selective layers (HSLs), self-assembled films offer distinct advantages, including superior optical transmittance, reduced parasitic absorption, and minimized series resistance. Initially, SAMs played an important functional role in field-effect transistors and light-emitting diodes [16]. Their subsequent integration into PSCs, particularly within inverted single-junction architectures, has driven remarkable advancements in device performance within just a few years [17]. Moreover, the application of SAMs has substantially accelerated the efficiency gains in perovskite tandem solar cells (TSCs) and enhanced the operational stability of large-area modules, advancements intrinsically linked to the unique molecular structures of SAMs. As highly ordered organic molecules, SAMs are typically composed of three parts: an anchoring group, a linkage, and a terminal group, and each functional component performs a distinct role depending on its chemical composition, as illustrated in Fig. 2a. The interaction between anchoring groups and metal oxides (such as ITO, FTO, and NiO_x_) can effectively modulate interfacial energy levels, thereby facilitating more efficient hole extraction. Moreover, enhanced hole extraction at the interface can be achieved by minimizing the energy offset between the highest occupied molecular orbital (HOMO) of the HTL and the valence band maximum of the perovskite (Fig. 2b) [18]. When SAMs with large dipole moments are formed on conductive electrodes, they can substantially shift the WF, thereby modulating carrier transport across the interface (Fig. 2c, d) [19]. The underlying mechanism is as follows: SAM molecules possess electronic asymmetry, wherein covalently bonded atoms or groups carry spatially separated partial positive and negative charges [20]. The electronegativity disparity between these atoms or groups generates a strong dipole moment, directed from the negative ends toward the positive ends [21, 22]. When these dipoles are assembled on conductive electrodes, they induce significant changes in the WF, thereby affecting carrier transport across the interface [23]. Moreover, by introducing linker groups and terminal groups, the physical properties of self-assembled films, such as wettability and energy levels, can be tailored to meet the requirements for PSC fabrication. The design of SAM molecules should adhere to key guidelines, including: (i) achievement of suitable energy-level alignment, (ii) ensuring regular and dense molecular packing, and (iii) establishing a strong interfacial interaction with both the substrate and the active layer. These factors are essential for achieving efficient charge transport, suppressing interfacial recombination, and improving device performance and stability.

#### Anchoring Groups

During device fabrication, the anchoring groups of vapor- and solution-processed SAMs spontaneously form chemical bonds with the surface of TCOs, thereby modulating the interfacial energy levels and facilitating efficient hole extraction [24]. Consequently, the adsorption efficiency of SAMs is critically governed by both the nature of the anchoring groups and the chemical characteristics of the substrate surface. For instance, Feng et al. [25] introduced a layer of –OH rich ITO nanoparticles (INPs) to provide abundant sites for SAM adsorption, thereby reinforcing their bonding strength and minimizing unnecessary hetero-contacts with the underlying ITO substrate. Different types of anchoring groups can induce unique binding configurations on target substrates, such as phosphoric acids, carboxylic acids, or silanes, among others, thereby serving as one of the criteria for classifying SAMs.

**Fig. 3:**
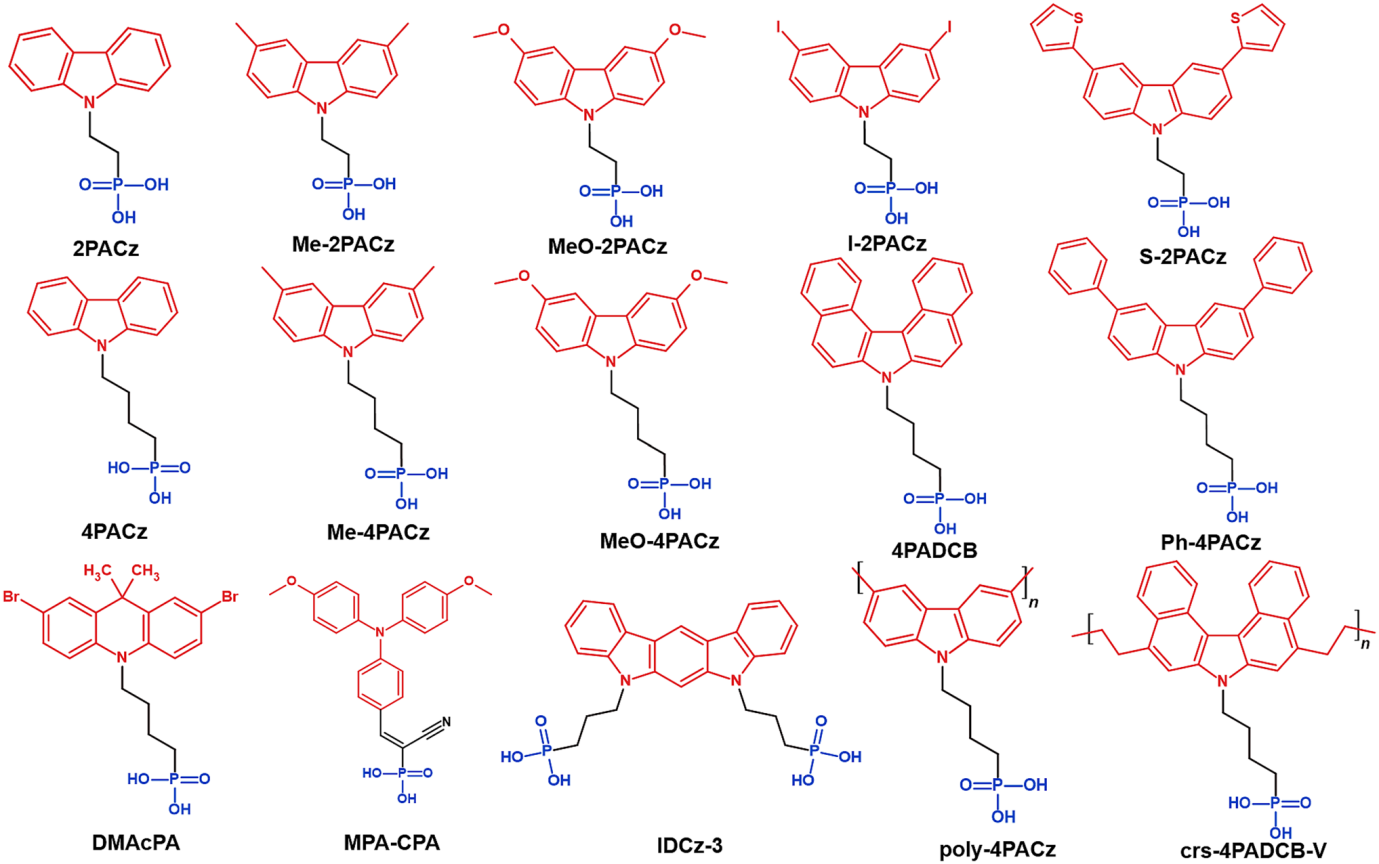
Molecular configurations of typical SAM with phosphonic acid anchoring groups

Figure 2e illustrates the anchoring mechanisms associated with different anchoring groups. Among these, SAMs featuring phosphonic acid anchoring groups have been most extensively utilized in PSCs. Figure 3 highlights representative molecular configurations of phosphate-based SAMs that are widely employed in PSC architectures. Although these SAM molecules differ in their molecular linker backbones and terminal functional groups, they typically exhibit a similar anchoring motif that enables robust attachment to TCO substrates. A phosphonic acid anchoring group, comprising two hydroxyl groups and an additional oxygen atom, enables versatile coordination with different metal atoms in TCOs. On TCO substrates, the formation of SAM generally proceeds through a sequential, multistep anchoring process [26]: (1) SAM molecules initially aggregate on the TCO surface through intermolecular interactions (such as hydrogen bonding between phosphonic acid groups), while their phosphonic termini establish secondary interactions with the substrate through hydrogen bonding or van der Waals forces, thereby forming a weakly physisorbed layer. (2) Subsequent thermal annealing induces dehydration–condensation reactions between surface hydroxyl groups on the TCO and phosphonic acid moieties, forming covalent P–O–M (M = metal) bonds. (3) Next, a rinsing step removes physically adsorbed species, leaving behind a chemically anchored monolayer. Covalent attachment of SAMs to metal oxide surfaces occurs through mono-, bi-, or tridentate coordination modes. Among these, monodentate binding proceeds via condensation of an anchoring –OH group with surface hydroxyls on the metal oxide, leading to the formation of strong P–O–M bonds [27]. In bidentate binding, a hydrogen atom is transferred to a surface hydroxyl group, facilitating a second condensation reaction. Similarly, owing to the presence of three oxygen atoms in the phosphonic acid anchoring group, tridentate binding configurations are also possible depending on the type and nature of metal oxide surface and the reaction conditions [28]. Nevertheless, the relative stability of these binding modes remains unresolved. Experimental studies have suggested that monodentate binding configurations are unstable, as they are prone to desorption or displacement by competing ligands in solution, ultimately comprising film integrity under operational stress [29, 30 ]. Conversely, theoretical investigations suggest that the most stable adsorption geometry involves coordination of the P=O group with the TCO surface, while the remaining two P–OH groups interact with surface hydroxyls through hydrogen bonding [31]. This indicates that even a nominally monodentate configuration can provide strong interfacial stabilization.

**Fig. 4:**
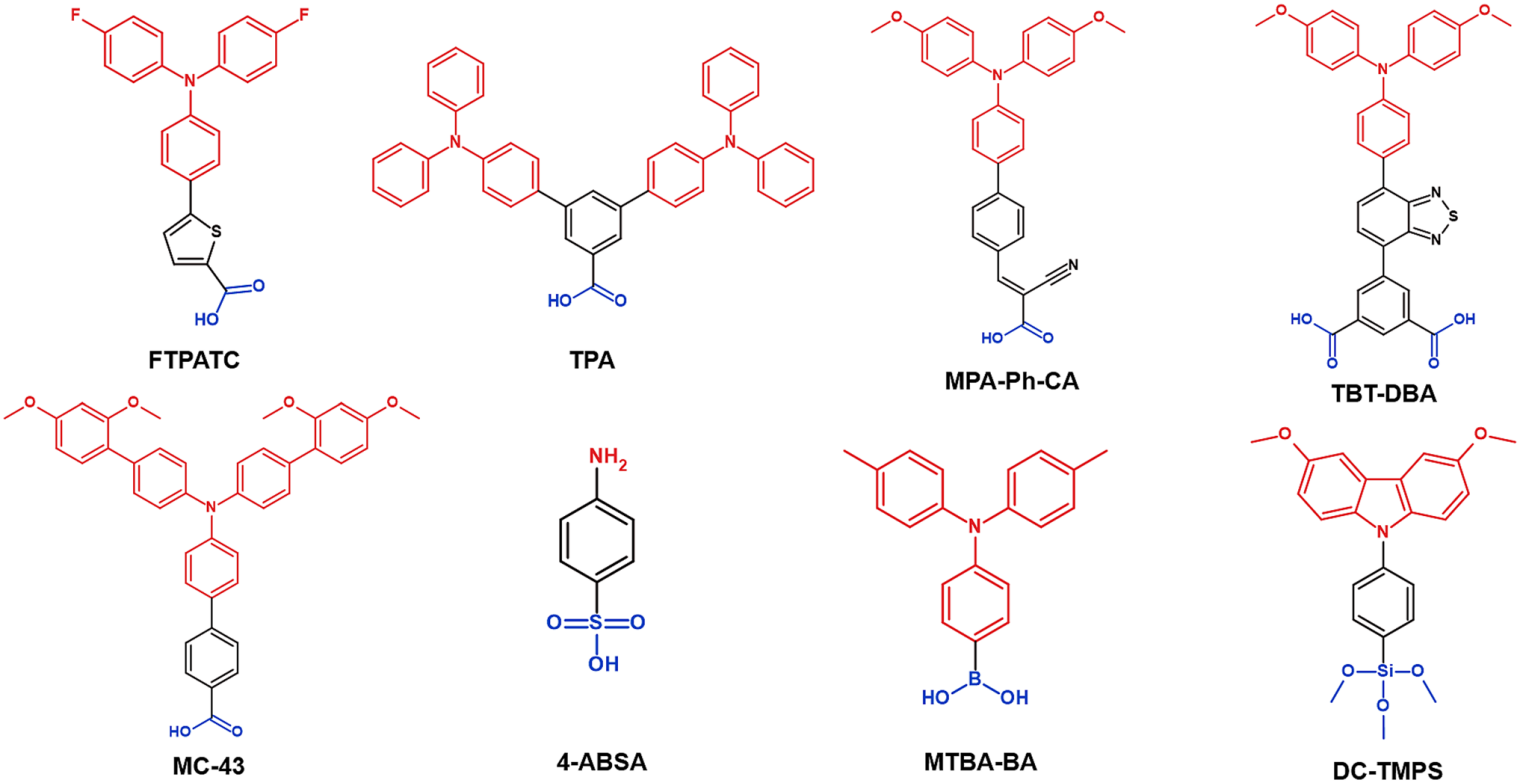
Molecular configurations of typical SAM with carboxylic acid and other different anchoring groups

Figure 4 presents several representative SAM molecules incorporating carboxylic acid and other anchoring groups that have recently been used as HTLs in PSCs. Different anchoring groups can significantly influence the adsorption behavior, molecular packing, and interfacial interactions between SAMs and TCO substrates. Among them, SAMs with carboxylic acid anchoring groups, featuring hydroxyl and oxygen atoms, follow a condensation pathway comparable to that of other anchoring motifs. They can form C–O–M covalent bonds on metal oxide surfaces through monodentate binding or achieve bidentate coordination by transferring a hydrogen atom to the surface hydroxyl group [28]. Zhang et al. [32] demonstrated that SAMs bearing carboxylate groups establish stronger interactions with perovskite films and yield better-oriented, more ordered monolayers compared to their phosphonate-based counterparts. The improved chemical affinity of carboxylate-anchored SAMs effectively reduces non-radiative recombination and enhances perovskite crystallinity. For example, Yalcin and colleagues [33] introduced 4,4″-bis(diphenyl amino)-1,1′:3′,1″-terphenyl-5′-carboxylic acid (TPA) and (4′-[bis(2′,4′-dimethoxybiphenyl-4-yl)amino]-biphenyl-4-carboxylic acid (MC-43) as potential alternatives to the widely utilized PEDOT:PSS for hole-selective contacts in PSCs. Consequently, the performance of device utilizing MC-43 increased to 17.5%. Similar bonding and anchoring mechanisms have also been observed in sulfonic-acid-based SAMs. Zhang et al. [34] employed a multifunctional 4-aminobenzenesulfonic acid (4-ABSA) interlayer to regulate energy-level mismatch between Ni^≥3+^ species at the NiO_x_/perovskite interface and Sn^2+^ in lead–tin (Pb–Sn) perovskites. This interfacial modification effectively reduced defect density and facilitated the growth of enlarged perovskite grains. Consequently, Pb–Sn PSCs achieved a PCE of 17.4%. Furthermore, the incorporation of the 4-ABSA interlayer markedly enhanced ultraviolet (UV) resistance and operational stability, retaining over 80% and 90% of the initial efficiency after 240 h of UV exposure and 480 h of 1-sun illumination, respectively. Li and co-workers [35] developed a series of hole-transporting SAMs incorporating diverse anchoring groups (–SO_3_H, –COOH, and –PO_3_H_2_) to systematically investigate the effect of bonding strength on monolayer quality and its correlation with the performance of p-i-n structured PSCs. Their study demonstrated that anchoring groups with stronger bonding strength accelerated SAM assembly promoted higher packing density and greater compactness, consequently enhancing charge collection while suppressing interfacial recombination losses. Nevertheless, Guo and his co-workers [36] found that the strong acidity of conventional anchoring groups in SAMs could be detrimental to device stability. To address this issue, they proposed, for the first time, the use of weakly acidic boronic acid as an alternative anchoring group to construct efficient SAM-based hole-selective contacts while mitigating ITO corrosion. The optimized boronic-acid-anchored hole-selective contact delivered a PCE approaching 23% with a high fill factor (FF) of 85.2%.

Unlike the aforementioned SAMs, those bearing silane anchoring groups (RSi_3_; X=Cl or OR) typically undergo a three-step anchoring process. First, the SAMs undergo hydrolysis on the substrate surface, transforming the three Si–X bonds into Si–OH groups. These hydroxyl groups then condense with –OH groups on the metal oxide surface to form Si–O–M bonds. Further, they interact with neighboring silanols to form Si–O–Si covalent bonds, eventually creating a cross-linked network on the substrate surface [37]. Tang et al. [31] demonstrated that achieving long-term operational stability in PSCs requires SAMs to remain densely packed beneath the perovskite layer throughout device operation. They discovered that SAMs anchored through hydrogen-bonded hydroxyl groups, rather than fully covalent attachment, are vulnerable to desorption when exposed to highly polar solvents present in the perovskite precursor. To address this challenge, they employed atomic layer deposition (ALD) technique to fabricate ITO substrates with fully covalent hydroxyl termination, ensuring robust SAM anchoring. In combination, trimethoxysilane-functionalized SAMs established strong tridentate binding to the substrate surface, significantly improving interfacial durability. Similarly, Soliman et al. [38] utilized cross-linkable organosilanes, n-propyltrimethoxysilane (PTMS) and (3-mercaptopropyl)trimethoxysilane (MPTMS), in conjunction with phosphonic acid-anchored [4-(3,6-dimethyl-9H-carbazol-9-yl)butyl]phosphonic acid (Me-4PACz) to construct self-assembled bilayers. The cross-linked organosilane network acted as a robust protective layer, preventing solvent-induced desorption of SAM and filling molecular voids, thereby generating a denser and more stable interface. Moreover, the thiol group in MPTMS strongly interacted with undercoordinated Pb^2+^ at the buried perovskite interface, further reducing defect density. Consequently, the devices achieved a high PCE of 24.9% with a T80 lifetime of 475 h under continuous 1-sun-equivalent illumination in air.

Overall, phosphonic acid anchoring groups typically exhibit the strongest interaction with TCO substrates, and this robust interfacial bonding contributes to denser molecular packing and suppressed interfacial non-radiative recombination. Moreover, the stronger anchoring effect often endows phosphonic acid-based SAMs with excellent interfacial stability. However, the relatively strong acidity of phosphonic acids can corrode both ITO substrates and perovskite layers, thereby accelerating interfacial degradation. By contrast, carboxylic acid anchoring groups usually exhibit weaker binding affinities to TCO surfaces, possibly leading to partial SAM desorption during device operation. Nevertheless, their weaker acidity and better chemical compatibility with perovskite precursors are beneficial for perovskite wetting, crystallization, and molecular ordering. Moreover, silane anchoring groups can not only form covalent Si–O–M bonds with substrates, but also generate cross-linked Si–O–Si networks, thereby significantly enhancing interfacial compactness, hydrolysis resistance, and resistance against solvent-induced SAM desorption. However, the existence of such cross-linked networks may also impede charge transport, resulting in relatively low charge extraction efficiency compared with the aforementioned SAMs.

#### Tailorable Linkers

**Fig. 5:**
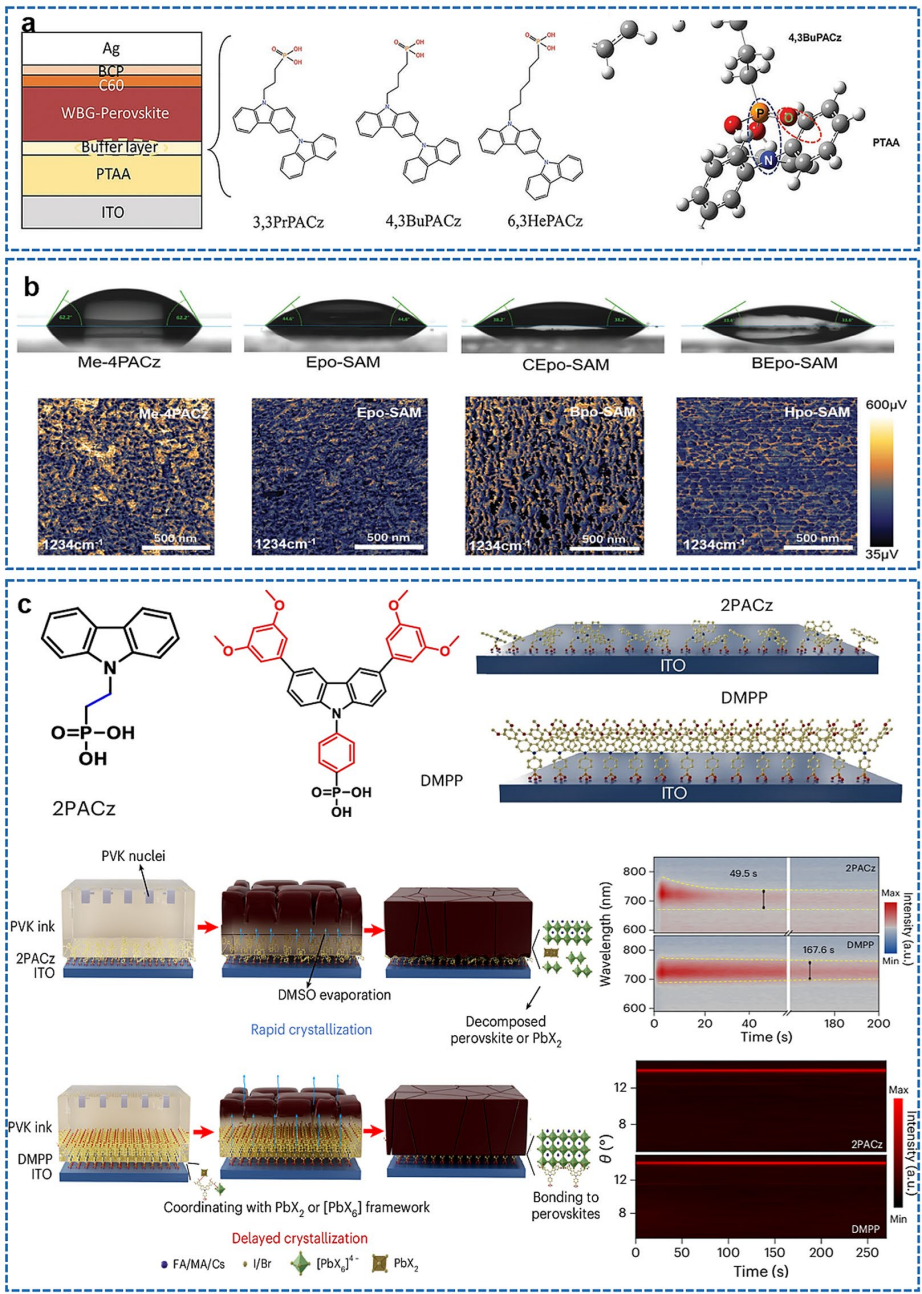
**a** Structure of device and molecular structures for surface modification and the chemical structure of 3,3PrPACz, 4,3BuPACz, and 6,3HePACz (left). Schematic diagram of the chemical interaction between PTAA and 4,3BuPACz (right) [40]. Copyright 2023 Wiley–VCH. **b** Perovskite contact angle tests (top) and photo-induced surface potential mapping at wavenumber of 1234 cm^−1^ on different po-SAM samples (bottom) [42]. Copyright 2023 Wiley–VCH. **c** Molecular structures of 2PACz and DMPP and a schematic diagram of the films they form on planar ITO (top). Schematic of the crystallization process of the perovskite film on 2PACz and DMPP substrates (bottom-left). In situ PL and XRD monitoring of PVK crystallization on 2PACz and DMPP substrates (bottom-right) [43]. Copyright 2025 Springer Nature Limited

Once effective anchoring is achieved, the length and intrinsic rigidity or flexibility of the spacer group dictate the specific molecular stacking patterns and geometric arrangement of the SAMs. In particular, longer alkyl chains exhibit stronger van der Waals interactions, generally leading to denser molecular packing and improved SAM ordering. Liu and Miao [39] reported that with the increase in the number of carbon atoms in n-alkyl phosphonic acids from 6 to 18, the arrangement of SAMs on the substrate surface transformed from disordered to ordered. Moreover, in inverted PSCs, longer alkyl chains could help release more stress within the overlying perovskite lattice. As illustrated in Fig. 5a, Bi and his co-workers [40] demonstrated that modifying PTAA with different SAMs revealed a clear trend: Increasing the alkyl chain length enhanced the quality of the overlying perovskite thin film by enlarging grain size and reducing pinhole density. In general, incorporation of SAM effectively suppresses non-radiative recombination and reduces valence-band offsets between the perovskite and HTL, thereby facilitating hole collection. Ramón’s group [41] reported a series of dipodal SAMs based on π-expanded indolo[2,3-a]carbazole motifs used as hole-selective contacts and systematically investigated the influence of spacer length. The SAM with a longer pentyl spacer (5CPICZ) delivered the highest PCE of 22.20%, slightly outperforming its propyl-spacer analogue (3CPICZ, 22.01%). Moreover, such spacers influence the charge transport properties at the interface.

As depicted in Fig. 5b, Zhao et al. [42] employed alkylphosphonic acids of varying chain lengths in a post-assembly process to fill surface voids, thereby forming dense po-SAM layers. They found that Br-EPA, characterized by its shorter alkyl chain and stronger interaction with perovskite precursor solutions, significantly improved perovskite wetting and device reproducibility. Therefore, for practical applications, an appropriate thickness must be ensured—sufficient to allow free carrier tunneling while simultaneously serving as a physical barrier. Therefore, SAM molecules with longer linkers promote the formation of denser SAM layers, whereas those with shorter linkers are more effective in enhancing perovskite wetting and facilitating uniform film formation. Therefore, optimizing linker length necessitates a careful balance between maintaining SAM compactness and ensuring perovskite wettability, both of which are critical for achieving peak device performance. Similarly, replacing the flexible butyl chains of carbazole-based SAMs with conjugated phenyl linkers was shown to significantly enhance their photostability [44]. Levine’s group [45] reported that in nPACz (*n* = 2, 4, 6) SAMs, increasing the alkyl spacer length led to a significant reduction in hole transport rates, thereby degrading device performance. However, the influence of spacer length-dependent variations in SAM tilt angle was not explicitly considered. Besides spacer length, linker conductivity has also been investigated. For instance, Zhao et al. [46] designed a series of SAMs in which the linker length and anchoring groups were kept constant while gradually extending the *π*-conjugated core. They demonstrated that enhanced π-conjugation within the SAM backbone significantly improved hole extraction efficiency. Zhang et al. [11] further demonstrated that extending molecular conjugation through aromatic linkers led to effective enhancement in both the photochemical and electrochemical stability of SAMs. The designed SAM, 3-(4′-(bis(4-methoxyphenyl)amino)-[1,1′-biphenyl]-4-yl)-2-cyanoacrylic acid (MPA-Ph-CA) featured a conjugated molecular structure that not only facilitated charge transport but also stabilized electron-rich arylamines through effective electron/charge delocalization. Moreover, it enabled convenient modulation of frontier orbital energy levels for interfacial band alignment. The incorporation of MPA-Ph-CA yielded more consistent device parameter distributions and substantially improved maximum power point (MPP) stability under operational light-soaking conditions. As a result, the inverted PSC achieved a PCE of 22.53% and maintained over 95% of its initial efficiency after 800 h of continuous one-sun irradiation. Moreover, SAMs featuring linkers with unique structural segments, such as heteroatoms, have also been extensively investigated. For example, Sheng’s group [47] harnessed the synergistic effects of extended conjugation and heteroatom incorporation by introducing MPA-Th-CA with a thiophene linker into PSCs. Leveraging its larger dipole moment, improved conductivity, and optimized energy-level alignment with the perovskite absorber, the resulting device attained a PCE of 25.53%. As presented in Fig. 5c, Zhang et al. [43] demonstrated that the structural and energetic incompatibility between conventional SAMs and perovskite precursor solutions resulted in uncontrolled crystallization kinetics, thereby inducing electronic defects and morphological degradation at the buried interface. To address this limitation, they engineered SAMs incorporating rigid conjugated linkers, which enhanced electrical conductivity and amplified dipole moments, thereby strengthening hole extraction. By using this strategy, single-junction PSCs achieved a PCE of 24.36%, while monolithic perovskite–silicon TSCs reached champion efficiencies of 33.86% (1 cm^2^) and 29.25% (16 cm^2^).

Overall, longer alkyl chains generally exhibit stronger van der Waals interactions, fostering denser and more ordered molecular packing within SAMs. Moreover, the flexibility of extended chain linkers helps relieve interfacial stress generated during perovskite film crystallization, thereby mitigating interfacial defects and film cracking. However, excessively long insulating alkyl chains can impede hole transport rates. By contrast, shorter linkers strengthen the interaction between SAMs and perovskite precursor solutions, thereby improving surface wettability, promoting uniform perovskite film formation, and enhancing device reproducibility, although their capability to optimize perovskite films remains comparatively limited. Furthermore, linkers incorporating rigid aromatic conjugated structures generally enhance the intrinsic conductivity of SAM molecules, thereby promoting more efficient interfacial hole extraction and reducing charge accumulation at the interface. Moreover, electron delocalization within conjugated structures enhances the photochemical and electrochemical stability of SAMs, thereby suppressing molecular degradation under prolonged illumination.

#### Functional Terminal Groups

SAM layers in PSCs are typically ultrathin, measuring only ~ 1–2 nm. Owing to their intrinsically low electrical conductivity, most SAM-based PSCs exhibit limited charge-collection efficiency. Moreover, as anchoring groups and linkers are often electrically insulating, terminal functional groups are deliberately engineered to possess semiconducting properties to facilitate efficient charge transport. The terminal groups should uniformly cover almost the entire substrate surface while remaining exposed. Since they are in direct contact with the perovskite layer, their selection is critical: the terminal groups govern the electronic coupling with the overlayer and thereby dictate the energy-level alignment between the SAM and the perovskite [48]. Therefore, commonly employed terminal groups in SAMs for PSCs include pyridines, thiols, ammonium cations, and cyano groups. These functionalities establish strong interactions with the inorganic [PbI_6_]^4−^ framework, thereby modulating the surface WF. Huang and co-workers [49] designed donor–acceptor SAMs incorporating oligomeric ether chains of varying lengths as terminal functional groups, yielding two new SAMs (EPA and MEPA) with enhanced bottom–up perovskite growth regulation and energy-level tuning capability. Oligoether-functionalized SAMs promoted ordered growth, high crystallinity, and favorable energy-level alignment at the top surface of perovskite films, enabling devices with a PCE of 25.50% and maintaining 90% of their initial efficiency after 1260h under ISOS-L-1 testing conditions. Polymeric carbazole phosphonic acid SAMs extend beyond conventional small-molecule systems, exhibiting robust hole extraction capability and pronounced suppression of interfacial recombination, thereby fostering stable perovskite/HTM interfaces. Ren et al. [50] addressed the sensitivity of carbazole phosphonic acid (PACz) HTMs toward layer thickness and substrate roughness by polymerizing PACz into a multifunctional polymeric derivative (Poly-4PACz). This polymer HTM exhibits excellent hole extraction capability; more importantly, Poly-4PACz demonstrates high conductance and thickness insensitivity on both ITO and FTO substrates, which is highly advantageous for scalable perovskite film deposition. Consequently, blade-coated p-i-n PSCs and modules attained impressive PCEs of 24.4% (6.84 mm^2^) and 20.7% (25.0 cm^2^), respectively. Similarly, Wang’s group [51] developed a polymeric HTM, Poly-DBPP, which exhibited higher conductivity, superior film uniformity, improved surface wetting, and suppressed interfacial recombination. Poly-DBPP-based PSCs delivered PCEs of 25.1% and 22.0% for blade-coated devices and large-area modules, respectively. During PSC fabrication, Wang, Liu, and co-workers [52] demonstrated that polymerized SAMs could effectively suppress the desorption of weakly adsorbed molecules during polar-solvent processing steps. To this end, they developed a novel SAM bearing *in-situ* cross-linkable moieties, 4-(5,9-diallyl-7H-dibenzo[c,g]carbazol-7-yl)butyl phosphonic acid (4PADCB-V), which was successfully cross-linked via UV irradiation. The resulting cross-linked SAM (crs-4PADCB-V) retained its structural integrity when exposed to polar solvents, mitigated interfacial defect formation, enhanced charge-carrier transport, and facilitated improved perovskite crystallization. Consequently, the devices demonstrated a champion PCE of 26.46% and retained 91% of their initial efficiency after 1000 h of continuous MPP tracking under illumination. Although –PO(OH)_2_ anchoring groups have been widely used in SAM design, their use raises concerns regarding potential ITO corrosion, which can trigger indium dissolution and compromise substrate conductivity [53].

**Fig. 6:**
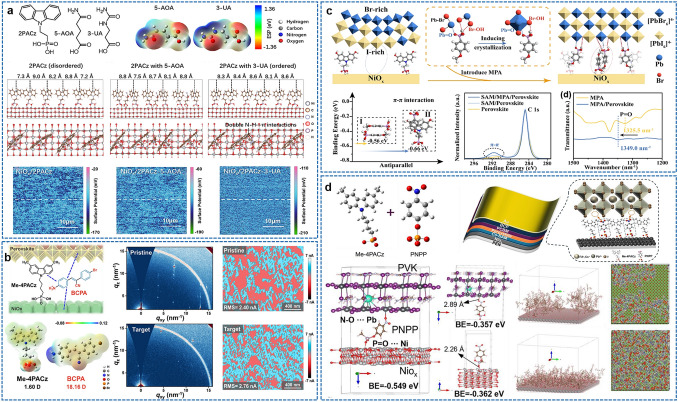
**a** Molecular structure and ESP of 3-UA and 5-AOA (top). DFT-based simulations of the self-assembly process (middle). KPFM images of 2PACz, 2PACz-5-AOA and 2PACz-5-AOA (bottom) [54]. Copyright 2025 Wiley–VCH. **b** Schematic diagram of BCPA-modified bi-hole transport layer and Molecular configurations of Me-4PACz and BCPA with calculated ESP (left). GIWAXS patterns of pristine and target Me-4PACz samples (middle). C-AFM images of pristine and target BHTL (right) [55]. Copyright 2025 Wiley–VCH. **c** Schematic diagram of the pristine film and the film with the MPA (top). Binding energy of the antiparallel structures of MPA with itself and MPA with MeO-2PACz calculated through DFT simulations (bottom-left). XPS spectra of C 1*s* for Perovskite, SAM/Perovskite, and SAM/MPA/Perovskite (bottom-middle). FTIR spectra of MPA and MPA/Perovskite (bottom-right) [56]. Copyright 2025 Elsevier. **d** Molecular structures of Me-4PACz and PNPP (top-left). Schematic diagram of the inverted device structure and the interaction role of Co-SAM with NiO_x_ and perovskite films (top-right). The rational design of molecules through DFT calculations and corresponding binding models of PVK, PNPP with NiO_x_ surface (bottom-left). DFT-optimized structure of Me-4PACz and Me-4PACz + PNPP on NiO_x_ surface (bottom-right) [57]. Copyright 2025 Wiley–VCH

**Fig. 7:**
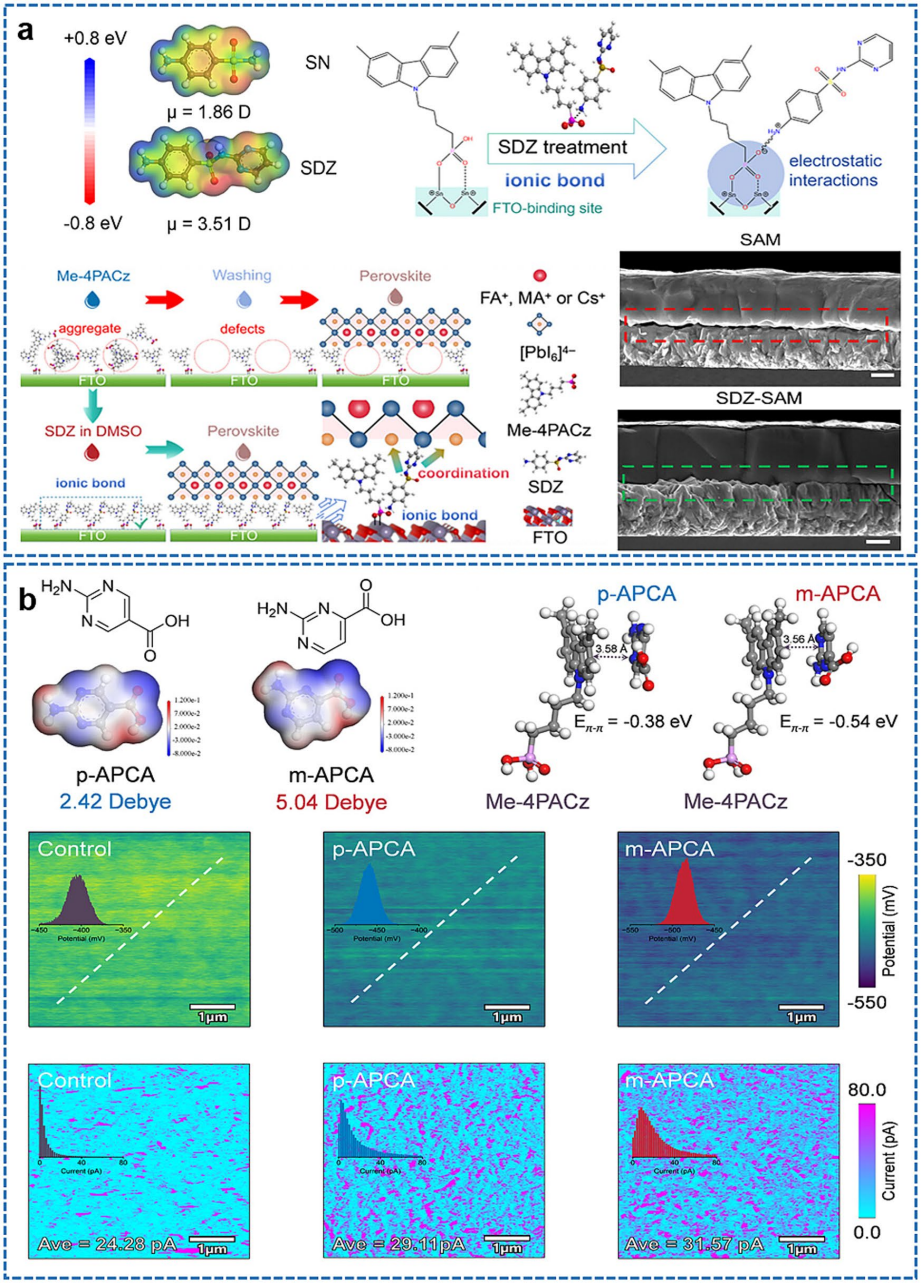
**a** Electrostatic potentials of SAs from DFT calculation (top-left). Schematic diagram of proton transfer and electrostatic interaction mechanism at the TCO/SAM interface (top-right) and Schematic diagram of EEAS mechanism (bottom-left). Cross-sectional SEM images of devices based on different SAMs (bottom-right) [58]. Copyright 2025 Wiley–VCH. **b** Chemical structure and ESP distribution of SAM molecules (top-left). The interaction configurations of p-APCA and m-APCA with Me-4PACz (top-right). KPFM (middle) and C-AFM (bottom) images of different substrates [59]. Copyright 2026 Wiley–VCH

In practical device fabrication, numerous studies have further demonstrated that incorporation of functional small-molecule additives can synergistically modulate SAM properties while simultaneously contributing to efficient hole transport. As illustrated in Fig. 6a, Zhang and co-workers [54] developed a co-assembled SAM incorporating 3-ureidopropionic acid (3-UA). The diamide terminal group in 3-UA strengthened chemical bonding with the buried Pb–I framework while simultaneously enhancing the structural ordering of the SAM, yielding compact and uniform monolayer assembly. The optimized SAM enabled a champion small-area device with a PCE of 25.3%, while demonstrating highly reproducible performance in devices with an active area of 1.02 cm^2^. After encapsulation, the devices maintained 92.8% and 91.2% of their initial PCE after 1500 h of aging at 85 °C and 1500 h of MPP tracking at 65 °C, respectively. As depicted in Fig. 6b, Zhou et al. [55] synthesized a novel multifunctional molecule with a highly polar architecture, (Z)-4-(2-(4-bromophenyl)-2-cyanovinyl)phenylamine (BCPA), which exhibits a ground-state dipole moment of 18.16 Debye, far exceeding that of the benchmark Me-4PACz (1.60 Debye). The cyano (–CN) group in BCPA coordinates with both Ni^x+^ and Pb^2+^, thereby modulating SAM anchoring. Its rigid planar geometry further promotes ordered packing of SAM via *π*–*π* interactions, collectively enhancing interfacial charge extraction. BCPA-modified HTLs significantly improved buried-interface quality by reducing trap density, enhancing crystallinity, and optimizing energy-level alignment. Similarly, Dong et al. [56] engineered a functional *π*-conjugated interfacial layer between the SAM and the perovskite by employing antiparallel-stacked 4-methoxyphenylphosphonic acid (MPA). Notably, *π*–*π* interactions among MPA molecules improved the SAM uniformity, while the phosphonic acid anchoring groups served as nucleation sites for perovskite crystallization, yielding uniform, high-quality perovskite films. The optimized buried interface facilitated efficient charge extraction, enabling a wide-bandgap (WBG, 1.68 eV) PSC to achieve a PCE of 22.91% with an open-circuit voltage (*V*_OC_) of 1.25 V, while retaining 90% of its initial performance after 2500 h of continuous 1-sun illumination without encapsulation (Fig. 6c). Furthermore, Wang et al. [57] integrated 4-nitrophenyl phosphate (PNPP) into Me-4PACz to improve the NiO_*x*_/perovskite interface. PNPP enhanced the surface uniformity and hydrophilicity of the NiO_*x*_/Me-4PACz layer, optimized perovskite crystal orientation, and effectively suppressed defect formation at both the NiO_*x*_ surface and the underlying perovskite, thereby markedly improving interfacial charge-transfer efficiency. Consequently, the efficiency of F-PSCs increased from 21.46% to 23.66%. Moreover, the F-PSCs retained 80% of their initial efficiency even after 10,000 bending cycles, a resilience attributed to improved stress distribution within the PVK and stronger interfacial adhesion (Fig. 6d). As presented in Fig. 7a, Wang and co-workers [58] utilized sulfadiazine molecules to deprotonate the phosphonic acid groups within SAMs, thereby generating phosphonate anions that enhanced electrostatic interactions with positively charged TCO substrates. This strategy strengthened the interfacial bonding between the SAM and the perovskite layer. Based on this approach, PSCs with a certified efficiency of 26.23% (1.036 cm^2^) were achieved. Furthermore, meta-substituted molecules containing asymmetric bifunctional groups on pyrimidine rings were introduced. These functional groups induced pronounced molecular polarization, thereby amplifying the overall dipole moment and reinforcing intermolecular *π*–*π* interactions. As illustrated in Fig. 7b, Wang et al. [59] introduced 2-aminopyrimidine-4-carboxylic acid (m-APCA) to mitigate SAM aggregation and promote uniform substrate coverage, thereby enabling PSCs with a PCE of 26.71%. In addition to pyrimidine derivatives, amide-based molecules have been extensively utilized for the modification of SAMs. For instance, Song’s group [60] introduced 4-(trifluoromethyl) benzamide (4-TB) into Me-4PACz to construct a synergistic SAM. The strong *π*–*π* stacking interaction between 4-TB and Me-4PACz suppressed molecular aggregation; moreover, the carbonyl functional group of 4-TB effectively passivated the undercoordinated Pb^2+^ defects at the buried interface. Based on this strategy, the 1.82 eV WBG PSC achieved a PCE of 19.44%, accompanied by a notably high *V*_OC_ of 1.33 V.

Overall, terminal groups are directly exposed at the SAM surface and in direct contact with the perovskite layer; therefore, their dipole moments and electronic structures play a decisive role in modulating the substrate WF. Terminal groups possessing strong dipole moments generally intensify the interfacial built-in electric field, thereby optimizing charge-carrier transport. In parallel, SAMs containing functional moieties such as pyridine, cyano, thiol, and ammonium moieties establish robust interactions with Pb^2+^ ions or the [PbI_6_]^4−^ framework, thereby effectively passivating buried interfacial defects and suppressing non-radiative recombination. Terminal groups featuring π-conjugated structures or cross-linkable moieties promote enhanced molecular ordering and structural integrity of the SAM layer through *π*–*π* stacking interactions or cross-linked networks. This structural reinforcement increases resistance to polar-solvent-induced erosion, mitigates SAM desorption, and enhances charge extraction efficiency. These characteristics are particularly advantages for improving interfacial stability and facilitating charge-transport efficiency during the fabrication of large-area devices. Conversely, terminal groups bearing hydrophilic or polar functionalities can optimize substrate wettability and facilitate the spreading behavior of perovskite precursor solutions; however, excessively strong polarity or acidity may accelerate interfacial chemical degradation.

### Preparation for SAMs

**Fig. 8:**
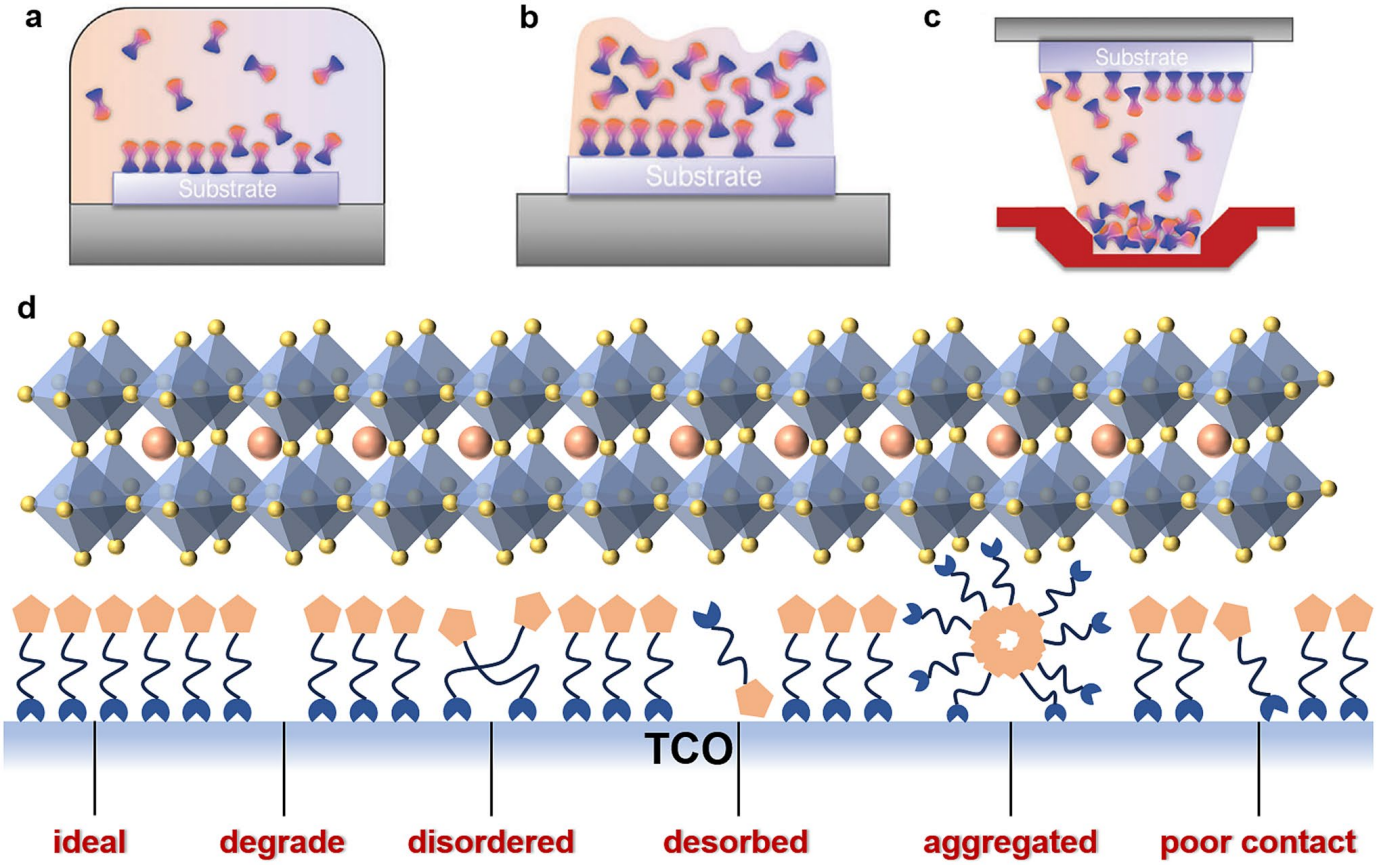
SAMs deposition methods [65]: **a** gas-phase self-assembly, **b** solution processing, **c** evaporation. Copyright 2024 Wiley–VCH. **d** The illustration of SAM assembly failure morphologies

To achieve strong binding between SAMs and metal oxides on the substrate, current SAM deposition techniques can be broadly classified into vapor-phase and solution-phase deposition techniques, as illustrated in Fig. 8a–c. One such vapor-phase approach is gas-phase self-assembly, in which the substrate is exposed to an atmosphere saturated with SAM molecules, facilitating the formation of an SAM [61]. A subsequent annealing step is typically performed to reinforce substrate bonding and eliminate excess, unbound molecules from the layer. Solution-processed SAMs frequently exhibit limited surface coverage and insufficient chemical anchoring on conductive substrates, particularly those with rough or flexible topographies, leading to compromised charge-transport efficiency and reduced long-term device stability. By contrast, vacuum thermal evaporation, a physical vapor deposition (PVD) technique, effectively circumvents these limitations by enabling highly uniform film deposition and fostering robust interfacial contact. Notably, Farag et al. [62] were the first to explore the use of PVD for fabricating uniform HTLs. In this approach, the substrate is placed inside a vacuum chamber, where SAM molecules are thermally evaporated to achieve sufficient bonding with the metal oxides on the substrate surface. This advancement holds pivotal significance for commercial photovoltaic production lines, as it enables the integration of SAM-based approaches into large-scale perovskite manufacturing. Zhang’s group [63] utilized vacuum thermal evaporation to deposit carbazole–phosphonic acid SAM thin films, systematically correlating molecular configuration with the resulting surface properties. They found that vacuum thermal-evaporated SAMs enhance surface energy, thereby accelerating perovskite nucleation kinetics, balancing film growth rates, and optimizing interfacial contact quality. Moreover, molecular configuration tailoring within the SAM backbone further improves buried-interface morphology and optimizes energy-level alignment, thereby promoting efficient charge transport while concurrently suppressing non-radiative recombination losses. As a result, devices incorporating vacuum thermal-evaporated SAMs achieved an impressive PCE of 25.47%. Nevertheless, during vacuum thermal evaporation, the random molecular orientations of the SAM indicate that not all phosphonic acid groups establish effective contact and anchoring with the ITO surface. This issue becomes more pronounced for larger molecules, and their thermal stability under evaporation temperatures remains unresolved [26].

Solution-phase deposition remains the predominant method for preparing SAMs, with representative techniques including spin-coating, dip-coating, and blade-coating. Among these, spin-coating is widely employed in laboratory practice as a rapid and simple process, wherein SAM molecules are dispensed onto a clean substrate at a controlled speed, followed by a thermal treatment to produce a uniform SAM [24]. Conversely, in dip-coating, the substrate is immersed into a SAM-containing solution to allow spontaneous adsorption [64], where effective chemisorption is achieved by tuning parameters such as solvent, concentration, and immersion time. Although vacuum deposition can yield higher surface coverage, the process is time-intensive, demands non-aggregating and thermally stable SAM precursors, and does not necessarily enhance monolayer ordering. Compared with spin-coating, dip-coating requires an additional solvent rinsing step to remove residual SAM molecules that are only weakly bound to the substrate [65]. Blade-coating technique offers a simple and highly efficient route for scalable solution processing; however, as substrate area increases, achieving uniform SAM coverage becomes a significant challenge, often evidenced by thickness variations between the leading and trailing edges of the film. Comparatively, spray-coating technique is more suitable for large-area substrates, but as a relatively complex technique, it generally requires precise control of the processing parameters to achieve high-quality coatings.

### Challenges of SAM Coating

#### Intrinsic Limitations of SAMs

**Fig. 9:**
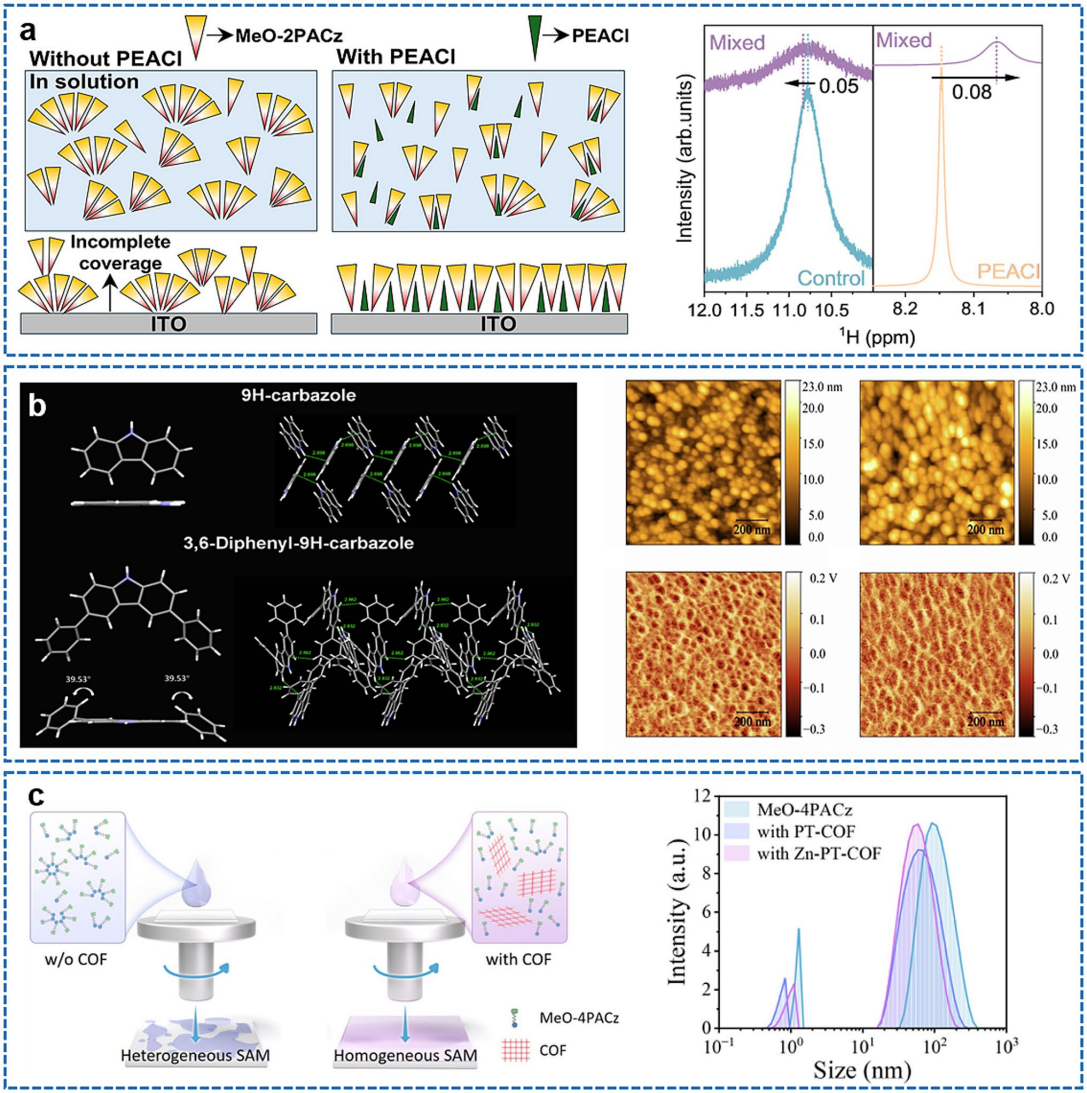
**a** Mechanism diagram of PEACl regulating SAM (left) and 1H NMR spectra of MeO-2PACz solution with and without PEACl, as well as the PEACl reference (right) [83]. Copyright 2026 American Chemical Society. **b** Molecular packing patterns of 2PACz and Ph-2PACz (left) and surface morphology (upper-right) and surface potentials (lower-right) of the hole-selective layers[84]. Copyright 2023 Elsevier Inc. **c** Schematic diagram of MeO-4PACz before and after COF co-assembly (left) and DLS patterns of MeO-4PACz solutions (right) [86]. Copyright 2025 Wiley–VCH GmbH

Although SAM-based PSCs have demonstrated impressive progress in PCE, it must be acknowledged that, relative to conventional HTMs, SAMs still suffer from degradation and wettability issues. These shortcomings frequently result in poor reproducibility and pronounced batch-to-batch variability [66], effects that become particularly severe under light and thermal stress [67]. One of the central challenges in SAM formation is the inherently complex task of constructing a high-density, closely packed molecular architecture [68]. Figure 8d illustrates that the instability of self-assembled films can originate from five main aspects, depending on the molecular structure of SAM and its interactions with different interfaces. Owing to the limited solubility of SAM molecules and their insufficient chemical affinity toward metal oxide surfaces, the interfacial bonds with TCO substrates are prone to disruption under external stress, resulting in molecular desorption [53]. Increasing the density of –OH groups on TCO surfaces increases the availability of anchoring sites for SAMs; however, it simultaneously leaves behind an excess of unreacted –OH groups. Formamidinium-based perovskites are susceptible to thermal- and moisture-induced degradation, generating acidic species [69]. Direct contact between alkaline TCO surfaces and acidic perovskites can intensify this degradation process. Furthermore, even covalent P–O–M linkages formed by phosphonic acid SAMs are not immune to degradation. Under humid conditions, hydrolytic cleavage of P–O bonds regenerates free hydroxyl groups on phosphorus, the reverse of dehydration condensation, eventually resulting in SAM desorption. Consequently, the SAM fabrication generally necessitates anhydrous or low-humidity environments to ensure interfacial stability [26]. Fei et al. [15] demonstrated that acidic protons from phosphonic acid, weakly coordinated to ITO surfaces, could accelerate iodide oxidation, formamidinium decomposition, and lead-ion reduction, processes that were further accelerated under elevated temperature and UV irradiation. Moreover, the intrinsic molecular structure of SAMs can also affect device performance. For instance, SAMs terminated with carbazole tend to undergo severe self-aggregation, driven by strong *π*–*π* interactions arising from the high planarity of the carbazole units [70]. This aggregation disrupts uniform molecular packing, resulting in heterogeneous coverage across the TCO surface [71, 72]. Moreover, at the SAM/perovskite interface, the interactions between SAM terminal groups and the perovskite lattice are easily disrupted during fabrication, thereby weakening interfacial passivation and contact, and ultimately accelerating perovskite degradation [53]. To overcome these challenges, several alternative strategies have been proposed. For instance, Zhao and co-workers [73] introduced a SAM (Py3) featuring a peri-fused polyaromatic core structure, which yielded superior carrier transport and selectivity compared with conventional heteroatom-substituted core frameworks. Benefiting from its relatively chemically inert and structurally rigid molecular contacts, the fabricated PSCs demonstrated an extended operational lifetime and a PCE of 26.1%. Pan et al. [74] constructed a sandwich-structured substrate consisting of aluminum oxide nanoparticles/SAM/aluminum oxide nanoparticles (Al_2_O_3_-NPs/SAM/Al_2_O_3_-NPs, denoted as ASA). This engineered architecture effectively filled microscopic grooves on the FTO surface, enabling the formation of a more uniform and compact SAM. Consequently, PSCs fabricated on ASA substrates achieved a PCE of 26.31%, accompanied by outstanding resistance to humidity and thermal stress. Similarly, Shi’s group [75] implemented dual buried-interface modification of e-beam-deposited NiO_x_ films using Me-4PACz as the SAM in combination with L-α-glycerylphosphorylcholine (GPC). The presence of GPC improved interfacial wettability and promoted more uniform SAM organization, thereby boosting the hole extraction capability of Me-4PACz as an HTL. Devices fabricated using this dual-modification strategy delivered a PCE of 23.31%. Trunong et al. [76] demonstrated that SAMs incorporating three phosphonic acid anchoring groups could adopt a face-on orientation on electrode surfaces, aligning toward adjacent layers and thereby enhancing interfacial charge extraction. Conversely, their monopodal counterparts generally adopt a more tilted molecular configuration, which diminishes the efficiency of charge transport. However, pendant acidic functional groups within these monolayers may also participate in undesirable electrochemical reactions with the perovskite layer, potentially compromising interfacial stability. Fei and co-workers [77] reported that acidic protons in phosphonic acid-based SAMs could accelerate iodide oxidation, promote formamidinium decomposition, and induce Pb^2+^ ion reduction. These degradation pathways are further exacerbated under elevated temperatures and UV irradiation, eventually resulting in device performance loss and instability. Besides, owing to the inherent amphiphilic nature of SAM molecules, their self-assembly mechanism resembles that of conventional surfactants [78]. For surfactants, monolayer formation generally occurs only when the concentration remains below the critical micelle concentration (CMC). Once the concentration exceeds the CMC, molecules preferentially aggregate into micellar structures, thereby suppressing the deposition of uniform monolayer [79]. Under conventional processing concentrations, SAM molecules are prone to pre-aggregation in solution, driven by hydrogen bonding, weak repulsive forces between molecules, and strong *π*–*π* stacking interactions. This aggregation favors the formation of micellar NPs rather than remaining as individually dispersed molecules [80]. These micelles can undergo further aggregation and, in some cases, precipitate from solution, which significantly compromises the storage stability of SAM inks in practical applications [81]. Moreover, such solution-phase aggregation can negatively impact molecular dispersion, film uniformity, and reproducibility of SAM-based HTLs, thereby influencing the final device performance and stability [80]. Therefore, introducing polar co-solvents or coordinating additives can improve the solubility of SAM molecules and modulate molecule–solvent interactions, thereby reducing molecular aggregation [51, 82]. For instance, Qi et al. [83] introduced the small-molecule PEACl, whose constituent cation–anion pair acted synergistically to disrupt the hydrogen-bonding network in the SAM solution and simultaneously broke up the large MeO-2PACz micelles (Fig. 9a). The resulting SAM film exhibited denser molecular packing, which facilitated enhanced hole mobility, alleviated residual stress, and promoted more favorable energy-level alignment with the perovskite layer. Additionally, structural modification of SAM molecules themselves can further mitigate undesirable self-aggregation. Typical strategies include incorporating molecules with larger steric hindrance, optimizing side-chain length, designing asymmetric molecular geometries, or introducing specific substituents intended to weaken intermolecular interactions. For example, Wang’s group [84] incorporated phenyl substituents with larger steric hindrance into the terminal groups, which attenuated π–π interactions and the associated undesirable aggregation, thereby facilitating self-assembly and yielding a more uniform surface potential, as presented in Fig. 9b. Liu and co-workers [85] co-assembled Me-4PACz with the multi-aromatic carboxylic acid 4,4′,4″-nitrilotribenzoic acid (NA) to improve the heterojunction interface. The incorporation of NA molecules improved the poor wettability and agglomeration behavior of the SAM layer, enabling the fabrication of devices with a PCE of 26.54%. Ma et al. [86] innovatively proposed incorporating covalent organic frameworks (COFs) into SAMs to alleviate molecular aggregation, as illustrated in Fig. 9c. Moreover, the COF facilitated charge transport, while its flexible hydrophilic side chains improved substrate wettability. Tan et al. [87] designed an asymmetric N-conjugated extended SAM architecture, where the two methyl groups on the asymmetric indene structure suppressed molecular aggregation and homogenized the spatial distribution of DMICPA, ultimately enabling PSCs with a PCE of 25.40%.

#### Morphology of SAM on TCO

**Fig. 10:**
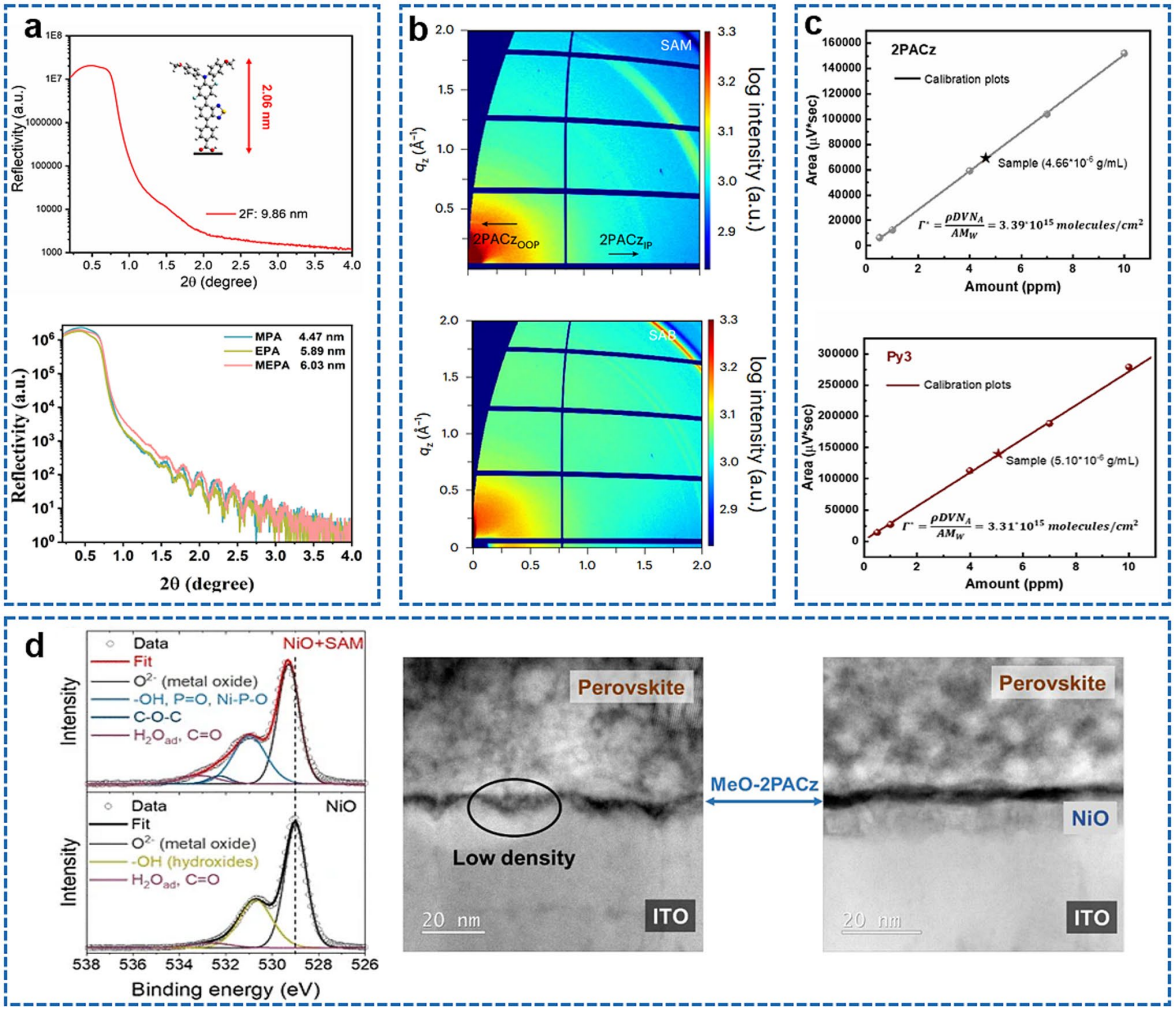
**a** XRR spectrum of the 2F grown on ITO glass (top) [13] and different SAMs grown on ITO/NiO_x_ substrate (bottom) [49]. Copyright 2023 The Author(s), under exclusive license to Springer Nature Limited and Copyright 2025 Wiley–VCH GmbH. **b** Two-dimensional GIWAXS patterns of 2PACz and SAB [89]. Copyright 2024 The Author(s). **c** Calibration plots and the determined concentration of 2PACz and Py3 in the UPLC measurements [73]. Copyright 2024 Springer Nature. **d** O 1*s* spectra of NiO and NiO + SAM samples (left). The HAADF images of a cross section of the two devices (right) [93]. Copyright 2021 The Authors

Notably, SAMs in PSCs are generally deposited on hydroxyl-rich metal oxides under solution-processing conditions; therefore, the assembly is influenced not only by molecular desorption but also by rapid solvent removal and intrinsic substrate heterogeneity, which together can yield more complex assembly configurations [88]. Recent experimental findings increasingly suggest that the actual assembly structures of SAM films may deviate from the idealized monolayer assembly model. For example, X-ray reflectivity analyses have in some cases revealed molecular-layer thicknesses approaching 100 nm, far exceeding the dimensions expected for a single monolayer (Fig. 10a) [13, 49]. Moreover, grazing-incidence wide-angle X-ray scattering patterns of SAM layers exhibit distinct diffraction features, revealing that these films are partially crystallized with long-range out-of-plane periodicity (Fig. 10b) [31, 89–91]. The existence of such long-range ordering suggests that the SAM layer cannot be confined to a strictly single-molecular monolayer; instead, it likely incorporates crystallized multilayers or partially aggregated molecular domains [92]. Furthermore, Tang et al. [73] utilized ultra-performance liquid chromatography to detach the adsorbed molecules from ITO substrates, subsequently dissolving them in methanol for quantitative analysis (Fig. 10c). The measured molecular density was determined to be approximately one order of magnitude greater than the expected density anticipated for an ideal monolayer configuration. Furthermore, high-angle annular dark-field imaging has directly visualized SAM interfacial layers with thicknesses in the range of 8–10 nm, substantially exceeding the molecular dimensions expected for a single monolayer (Fig. 10d) [31, 76, 93]. Tang et al. [31] investigated the evolution of molecular coverage by X-ray photoelectron spectroscopy and found that the conventionally adopted rinsing procedure fails to fully eliminate weakly adsorbed molecules.

Moreover, different TCO substrates possess distinct surface hydroxyl densities, surface energies, and chemical coordination environments, all of which critically modulate the anchoring behavior of SAMs. Different TCO substrates exhibit unique physicochemical characteristics that can strongly influence SAM assembly behavior. For example, on rough substrates such as FTO or textured silicon, the heterogeneous surface morphology may leave locally uncovered pinholes within the monolayer [94]. By contrast, deprotonation reactions can occur at the AZO/perovskite interface, leading to perovskite decomposition [95]. Kralj et al. [96] systematically compared amorphous and polycrystalline Sn-doped ITO substrates before and after anchoring with 2PACz-SAMs and NiO_x_/2PACz-SAMs, revealing that the microstructural characteristics of the TCO play a decisive role in governing the magnitude of the WF shift induced by SAM anchoring. Therefore, tuning the microstructure of TCOs is critical not only for realizing high-mobility, broadband transparent electrodes, but also for achieving a uniform WF distribution when interfaced with hole transport SAMs. Tang et al. [31] employed ALD to fabricate ITO substrates with fully covalent hydroxyl-terminated surfaces for SAM anchoring, thereby strengthening SAM binding and effectively mitigating SAM desorption induced by the highly polar solvents in perovskite precursor solutions. Nickel oxide (NiO_x_) is the most widely used HTL for high-performance inverted PSCs. However, dangling bonds on the NiO_x_ surface generate interfacial defect sites, hinder charge-carrier extraction, and promote interfacial carrier recombination [97]. Therefore, post-treatment of NiO_x_ using SAMs has also been extensively investigated. For example, Li et al. [98] utilized the SAM molecule (4-(3,11-dimethoxy-7H-dibenzo[c,g]carbazol-7-yl)butyl)phosphonic acid (MeO-4PADBC) to improve and stabilize the NiO_x_/perovskite interface, thereby enhancing surface coverage and delivering a certified PCE of 25.6%. Overall, different TCO substrates possess distinct surface hydroxyl densities, surface energies, roughness, and chemical coordination environments, which can strongly influence the assembly behavior of SAMs. Variations in substrate surface chemistry can lead to significant differences in SAM anchoring strength and interfacial bonding configurations. Differences in surface roughness and hydroxyl distribution simultaneously influence molecular packing density, film uniformity, and defect generation within the SAM layer. Additionally, substrate-dependent intermolecular interactions may induce variations in molecular orientation, including tilt angle, upright configuration, and π–π stacking behavior, which in turn modulate the surface WF and interfacial energy-level alignment, ultimately governing charge extraction and overall device performance.

## Application of SAMs as HTL

### High-Efficiency Perovskite Solar Cells

SAMs have garnered increasing attention as HTLs in high-efficiency inverted PSCs, owing to their potential to enhance interfacial energy alignment, passivation, and charge extraction, collectively enabling record device efficiencies. The wettability, adsorption capability, and compactness of SAMs critically influence perovskite film quality and defect formation at the perovskite–substrate interface, which in turn govern both the efficiency and stability of the device.

**Fig. 11:**
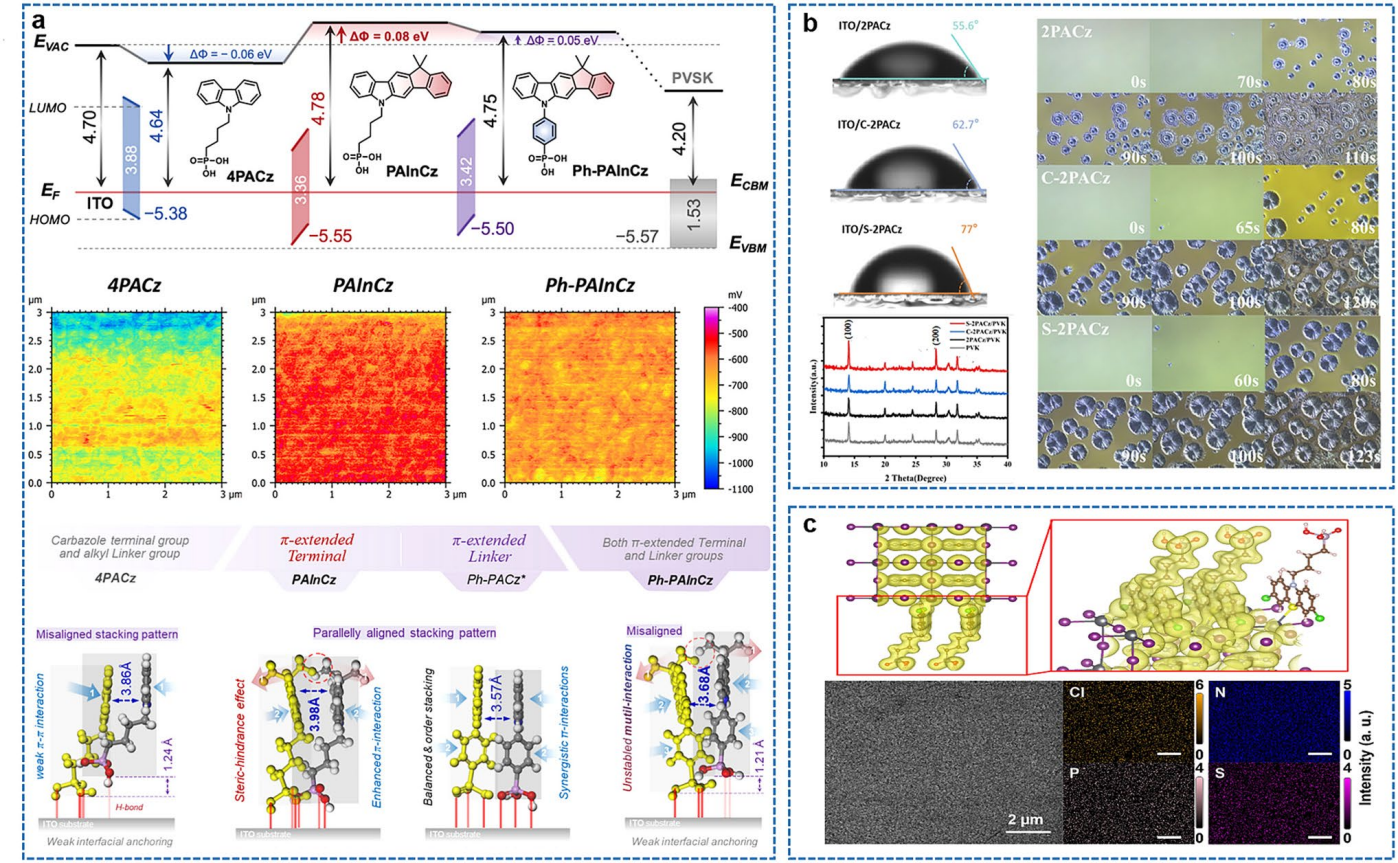
**a** Energy-level diagram (top). Visualized KPFM mapping images of ITO/SAM/PVK films (middle). Proposed conception and calculated intermolecular distance based on the molecular dimer of 4PACz, PAInCz, and Ph-PAInCz (bottom) [99]. Copyright 2025 Wiley–VCH. **b** Water contact angles (left-top), X-ray diffraction patterns of perovskite films grown (left-bottom), and optical microscopy images of growth kinetics of the perovskite precursor solutions deposited on three different SAM/ITO substrates (right) [100]. Copyright 2024 The Royal Society of Chemistry. **c** Interfacial orbital distribution of TDPA-Cl and perovskite from the DFT calculation. (top). SEM image and its corresponding Cl (orange), N (blue), P (pink), and I (purple) element EDS mapping of the TDPA-Cl SAM on ITO (bottom) [101]. Copyright 2024 American Chemical Society

In pioneering studies, Zhang and co-workers [99] designed a series of SAMs featuring an indenocarbazole (InCz) terminal group. The InCz-terminated phosphonic acid (PAInCz) strengthened π-interactions, fostering a densely packed and highly ordered molecular arrangement. Moreover, the incorporation of dimethyl substituents introduced steric hindrance that further improved film quality, resulting in excellent interfacial contact and energy-level alignment. Devices incorporating this SAM achieved a champion PCE of 25.51%, while exhibiting outstanding operational stability, maintaining over 85% of their initial efficiency after 500 h of continuous light soaking (AM 1.5G) and thermal stress at 65 °C (Fig. 11a). Wang et al. [100] synthesized S-2PACz, which passivated bulk defects in perovskite films through electrostatic interactions between undercoordinated Pb^2+^ centers and the sulfur atoms of its thiophene group. This interaction effectively reduced intergranular defects, mitigated charge recombination, and improved crystallization dynamics (Fig. 11b). Li and co-workers [101] designed an SAM, TDPA-Cl, by introducing chlorinated phenothiazine as the headgroup and linking it with the anchoring phosphonic acid through a butyl chain, as illustrated in Fig. 11c. This SAM was simultaneously applied in both organic solar cells and PSCs, exhibiting improved processability and reproducibility. Su et al. [102] developed a novel SAM material, 4-(3,6-di-tert-butyl-9H-carbazol-9-yl)butyl phosphonic acid (tBu-4PACz), and employed it in combination with MeO-2PACz as a co-HTL. The bulky tert-butyl substituents in tBu-4PACz introduced significant steric hindrance that prevented undesirable molecular aggregation and promoted better film conformality. Consequently, the perovskite films deposited on the co-SAM layer exhibited enhanced morphological quality, facilitating more efficient hole extraction and suppressing interfacial recombination losses. Therefore, the inverted PSCs based on tBu-4PACz achieved a certified PCE of 26.21% and retained over 94.7% of their initial efficiency after 500 h of MPP tracking under one-sun illumination. Wu and his colleagues [103] synthesized and structurally tailored IDCz-3 by repositioning its two nitrogen atoms to optimize the molecular dipole moment and enhance π-stacking interactions. This modification facilitated efficient hole extraction while suppressing electron back-transfer, enabling the PSC to achieve a PCE of 25.15%. Moreover, the unencapsulated devices exhibited exceptional ambient stability, sustaining nearly their initial performance even after 1800 h of storage. Similarly, Dai and his group [104] employed an iodine-terminated carbazole-based SAM (I-2PACz), which significantly enhanced the mechanical adhesion at the interface. The strengthened interfacial adhesion suppressed the nucleation and propagation of voids and cracks during PSC operation, allowing the device to retain 96% of its initial efficiency after 1000 h of continuous illumination under maximum power output. Moreover, SAMs also play vital roles in various perovskite optoelectronic device architectures. For instance, Wang et al. [105] employed [2-(9H-carbazol-9-yl)ethyl] phosphonic acid (2PACz) as an HTL for inorganic CsPbI_3_ PSCs. Leveraging the strong chemical coordination between its phosphonic acid anchoring groups and the FTO substrate, 2PACz establishes intimate interfacial contact and favorable energy-level alignment with the perovskite layer. This interfacial synergy promotes enhanced film quality, enables more efficient interfacial charge extraction, suppresses charge recombination, and reduces voltage losses. Consequently, the device achieved a champion PCE of 18.89% along with improved operational stability. Ilicheva et al. [106] presented the first demonstration of an SAM implementation enabling double-side passivation in p-i-n PSCs. The fluorinated SAM, 5-(4-[bis(4-fluorophenyl)amino]phenyl)thiophene-2-carboxylic acid (FTPATC), was incorporated at the hole transport interface, effectively alleviating interfacial barriers and lattice strain within the absorber. On the electron-transporting side, FTPATC interacted with A-site cations (Cs^+^, FA^+^), generating interfacial dipoles that compensated for defects. This dual-interface passivation delivered a PCE of 22.2%, while significantly enhancing operational durability, retaining 88% of its initial efficiency after 1680 h of continuous illumination (1 sun, 65 °C, ISOS-L-2), while also demonstrating robust thermal stability at 90 °C. Fürrer and colleagues [107] utilized drift–diffusion simulations to reveal that decreasing the WF of ITO by 0.2 eV could increase the efficiency of ETL-free PSCs by more than 30%. Consequently, naphthalene-imide SAMs were incorporated into ETL-free devices, where their strong chemical and thermal robustness substantially improved device stability. The pyridine-functionalized naphthalene diimide carboxylic acid-based device demonstrated a T80 operational lifetime exceeding 800h in air at 85 °C, coupled with enhanced stabilized power output attributed to the SAM-modified electron contact. Cho et al. [108] integrated MeO-2PACz-SAMs into Sn-based PSCs, wherein the P–O– anchoring groups electrostatically coupled with positively charged sulfur species (S^2+^) in PEDOT:PSS through interfacial deprotonation of phosphonic acid, thus modulating electrode WF. Furthermore, coordination between methoxy (–OCH_3_) oxygen donors and Sn-halide octahedra partially suppressed Sn^2+^ oxidation and reduced perovskite defect density, further enhancing device performance and operational stability. Feeney et al. [109] highlighted the critical role of substrate interfaces in co-evaporated PSCs. They employed SAM-HTLs for co-evaporated perovskite absorbers and revealed that the exposed phosphonic acid groups exert a significant influence on both the initial and final crystallographic phases of the co-evaporated perovskites. The surface interaction, governed by hydrogen bonding with interfacial iodine, enhances formamidinium iodide adsorption and induces persistent changes in perovskite structure, thus stabilizing bulk α-FAPbI_3_ through a hypothesized kinetic trapping mechanism. Table 1 summarizes representative SAM-based inverted PSCs along with their photovoltaic parameters, revealing that phosphonic acid anchoring groups consistently deliver the most well-balanced photovoltaic performance. In recent years, most high-efficiency devices have been based on phosphonic acid SAM-HTLs, a trend largely attributed to the stronger binding interaction between phosphonic acid groups and TCO substrates, resulting in better molecular ordering. By contrast, carboxylic-acid-based SAMs can still deliver relatively high FF values, yet their *V*_OC_ values are generally lower than those achieved with phosphonic acid-based SAMs. This disparity likely arises because the weaker polarity of carboxylic acid groups imposes less detrimental influence on perovskite film formation, while their relatively weak anchoring strength may lead to interfacial instability and less efficient charge extraction. Nevertheless, the performance of PSCs is not dictated solely by the SAM type, but is also strongly influenced by parameters such as perovskite composition, passivation layers, and device architecture.

**Table 1:** Typical SAM-based inverted PSCs and their champion photovoltaic parameters in recent years, where *V*_*OC*_ refers to the open-circuit voltage,* J*_*SC*_ refers to the short-circuit current density, and FF refers to the fill factor

SAMs	Anchoring group	Architecture	PCE (%)	*V*_oc_* (V)*	*J*_sc_* (*μA cm^−2^)	FF (%)	Refs.
2PACz	PO_3_H_2_	FTO/2PACz/CsPbI_3_/PCBM/BCP/Ag	18.89	1.168	20.51	78.85	[105]
3C-Ph	PO_3_H_2_	ITO/NiO_*x*_/SAM/Cs_0.18_FA_0.82_Pb(I_0.8_Br_0.2_)_3_/PEAI/PC_61_BM/BCP/Ag	21.59	1.230	21.53	81.52	[110]
PAInCz	PO_3_H_2_	ITO/SAM/Rb_0.05_Cs_0.05_MA_0.05_FA_0.85_Pb(I_0.95_Br_0.05_)_3_/PDADI/C_60_/BCP/Ag	25.51	1.191	25.47	84.08	[99]
5CPICZ	PO_3_H_2_	ITO/SAM/Cs_0.05_(FA_0.95_MA_0.05_)_0.95_Pb(I_0.95_Br_0.05_)_3_/PC_60_BM/BCP/Au	22.20	1.146	23.54	82.40	[41]
CbzBT	PO_3_H_2_	ITO/SAM/Cs_0.05_MA_0.15_FA_0.80_PbI_3_/C_60_/BCP/Ag	23.93	1.155	24.67	83.94	[111]
CzTh	PO_3_H_2_	ITO/SAM/Cs_0.05_(FA_0.92_MA_0.08_)_0.95_Pb(I_0.92_Br_0.08_)_3_/C_60_/BCP/Ag	23.45	1.149	24.58	83.06	[112]
E-CbzBT	PO_3_H_2_	ITO/SAM/perovskite/PEABr/PCBM/BCP/Ag	25.15	1.160	25.67	84.13	[113]
I-2PACz	PO_3_H_2_	ITO/SAM/Cs_0.05_FA_0.85_MA_0.10_Pb(I_0.97_Br_0.03_)_3_/LiF/C_60_/BCP/Ag	25.39	1.163	25.43	85.80	[104]
MeO-BTBT	PO_3_H_2_	ITO/SAM/Cs_0.05_MA_0.15_FA_0.80_PbI_3_/C_60_/BCP/Ag	24.53	1.160	24.87	85.28	[114]
Me-PhpPACz	PO_3_H_2_	ITO/SAM/Cs_0.2_FA_0.8_PbI_1.8_Br_1.2_/C_60_/BCP/Cu	26.17	1.189	25.37	86.79	[44]
MeS-4PACz	PO_3_H_2_	FTO/SAM/Perovskite/PDAI_2_/C_60_/BCP/Ag	25.13	1.144	25.73	85.37	[115]
m-PhPACz	PO_3_H_2_	FTO/SAM/Cs_0.05_MA_0.1_FA_0.85_PbI_3_/PCBM/C_60_/BCP/Ag	26.20	1.180	25.80	85.40	[116]
S-2PACz	PO_3_H_2_	ITO/SAM/Cs_0.2_FA_0.8_PbI_3_/C_60_/BCP/Ag	18.65	1.050	22.70	78.65	[100]
tBu-4PACz	PO_3_H_2_	ITO/SAM/FA_0.84_MA_0.11_Cs_0.05_Pb (I_0.987_Br_0.013_)_3_/EDAI_2_/C_60_/BCP/Ag	26.25	1.170	25.94	86.42	[102]
Spiro-S	PO_3_H_2_	ITO/SAM/Perovskite/PCBM/BCP/Ag	25.75	1.170	26.12	84.49	[117]
IDCz-3	PO_3_H_2_	FTO/SAM/Cs_0.05_FA_0.85_MA_0.1_PbI_3_/PI/C_60_/BCP/Ag	25.15	1.160	25.59	84.74	[103]
TPA2P	PO_3_H_2_	ITO/SAM/Cs_0.05_FA_0.95_PbI_3_/C_60_/BCP/Ag	26.11	1.180	25.99	85.03	[118]
Poly-DBPP	PO_3_H_2_	ITO/SAM/MA_0.7_FA_0.3_PbI_3_/C_60_/BCP/Cu	25.10	1.180	25.70	82.90	[51]
crs-4PADCB-V	PO_3_H_2_	FTO/SAM/Cs_0.05_FA_0.85_MA_0.1_PbI_3_/C_60_/BCP/Cu	26.46	1.194	25.68	86.29	[52]
4,3HePACz	PO_3_H_2_	ITO/PTAA/SAM/FA_0.8_Cs_0.2_PbI_1.8_Br_1.2_/C_60_/BCP/Ag	16.57	1.175	17.98	78.44	[40]
4-PhCz	PO_3_H_2_	ITO/SAM/Cs_0.17_FA_0.83_Pb(I_0.80_Br_0.20_)_3_/C_60_/BCP/Ag	22.53	1.273	21.27	83.21	[119]
BCBBr-C4PA	PO_3_H_2_	ITO/SAM/FA_0.8_Cs_0.2_Pb(I_0.6_Br_0.4_)_3_/C_60_/ALD-SnO_2_/Cu	18.63	1.286	17.54	82.61	[120]
A7HPA	PO_3_H_2_	ITO/SAM/Cs_0.05_FA_0.8_MA_0.15_PbI_2.29_Br_0.70_/PI/C_60_/BCP/Ag	23.41	1.260	21.82	85.15	[121]
*β*-NapPAPT	PO_3_H_2_	ITO/SAM/Cs_0.05_FA_0.8_MA_0.15_Pb(I_0.75_Br_0.25_)_3_/LiF/C_60_/BCP/Ag	21.86	1.212	20.93	86.18	[122]
2PhPA-CzH	PO_3_H_2_	ITO/SAM/Cs_0.05_FA_0.8_MA_0.15_Pb(I_0.75_Br_0.25_)_3_/perovskite/LiF/C_60_/BCP/Ag	22.83	1.238	21.27	86.70	[123]
4PABCz	PO_3_H_2_	ITO/SAM/Cs_0.05_(FA_0.98_MA_0.02_)_0.95_Pb(I_0.98_Br_0.02_)_3_/PDI/PCBM/BCP/Ag	26.08	1.20	25.75	84.54	[71]
4PA-Spiro	PO_3_H_2_	ITO/SAM/Cs_0.05_MA_0.05_FA_0.9_PbI_3_/PCBM/BCP/Ag	25.28	1.160	25.86	84.20	[124]
A-4PADCB	PO_3_H_2_	ITO/SAM/Cs_0.05_(FA_0.98_MA_0.02_)_0.95_Pb(I_0.98_Br_0.02_)_3_/PEAI/C60/BCP/Ag	25.05	1.186	25.39	83.14	[125]
Poly-DCPA	PO_3_H_2_	ITO/HTL/MA_0.7_FA_0.3_PbI_3_/C_60_/BCP/Cu	24.90	1.170	25.40	83.60	[126]
MeO-PhPACz	PO_3_H_2_	ITO/ALD-NiO_*x*_/SAM/Perovskite/C_60_/BCP/Ag	21.74	1.070	23.74	84.43	[127]
MeO-2PACz	PO_3_H_2_	ITO/PEDOT:PSS/SAM/PEA_0.15_FA_0.75_MA_0.10_SnI_2_Br/PCBM/BCP/Ag	12.16	0.930	16.60	78.76	[108]
MeO-4PADCB	PO_3_H_2_	ITO/NiO_x_/SAM/Perovskite/CF_3_-PEAI-MAI/C_60_/BCP/Ag	25.60	1.190	25.40	84.60	[98]
TDPA-Cl	PO_3_H_2_	ITO/SAM/Cs_0.05_MA_0.15_FA_0.80_PbI_3_/C_60_/BCP/Cu	22.40	1.151	23.90	81.40	[101]
FTPATC	COOH	ITO/SAM/Cs_0.2_FA_0.8_PbI_2.93_Cl_0.07_/SAM/C_60_/BCP/Cu	22.23	1.130	22.70	86.66	[106]
2,7CZ-FV	COOH	ITO/SAM/Cs_0.05_FA_0.93_MA_0.02_Pb(I_0.95_Br_0.05_)_3_/C_60_/BCP/Ag	24.17	1.131	25.48	83.86	[128]
9CAA	COOH	ITO/SAM/Cs_0.1_FA_0.6_MA_0.3_Pb_0.5_Sn_0.5_I_3_/C_60_/BCP/Ag	23.10	0.890	32.80	79.00	[32]
HC-A4	COOH	ITO/NiO_*x*_/SAM/CsFAMAPb(IBr)_3_/C_60_/BCP/Ag	20.00	1.050	23.34	81.80	[129]
TBT-BA	COOH	ITO/NiO_*x*_/SAM/Cs_0.04_(FA_0.96_MA_0.04_)_0.96_Pb(I_0.96_Br_0.04_)_3_/PEAI/PCBM/BCP/Ag	24.80	1.190	24.90	83.70	[130]
XS13	COOH	ITO/NiO_*x*_/SAM/FA_0.83_Cs_0.17_Pb(I_0.6_Br_0.4_)_3_/PEAI/PCBM/BCP/Ag	19.20	1.260	18.25	83.74	[131]
N719	COOH	ITO/SAM/Cs_0.10_FA_0.90_PbI_3_/C_60_/BCP/Ag	23.80	1.150	25.50	81.20	[132]
MEPA	COOH	ITO/NiO_*x*_/SAM/Perovskite/PEAI/C_60_/BCP/Ag	25.50	1.170	25.63	85.04	[49]
DC-TMPS	Si(OMe)_3_	ITO/SAM/Cs_0.05_(FA_0.95_MA_0.05_)_0.95_Pb(I_0.95_Br_0.05_)_3_/PEAI/C_60_/ZnO/Au	23.20	1.170	24.90	80.00	[31]
MTPA-BA	BO_2_H_2_	ITO/SAM/Cs_0.05_(FA_0.95_MA_0.05_)_0.95_Pb(I_0.95_Br_0.05_)_3_/PEAI/PCBM/C_60_/BCP/Ag	22.62	1.140	23.24	85.20	[36]

**Fig. 12:**
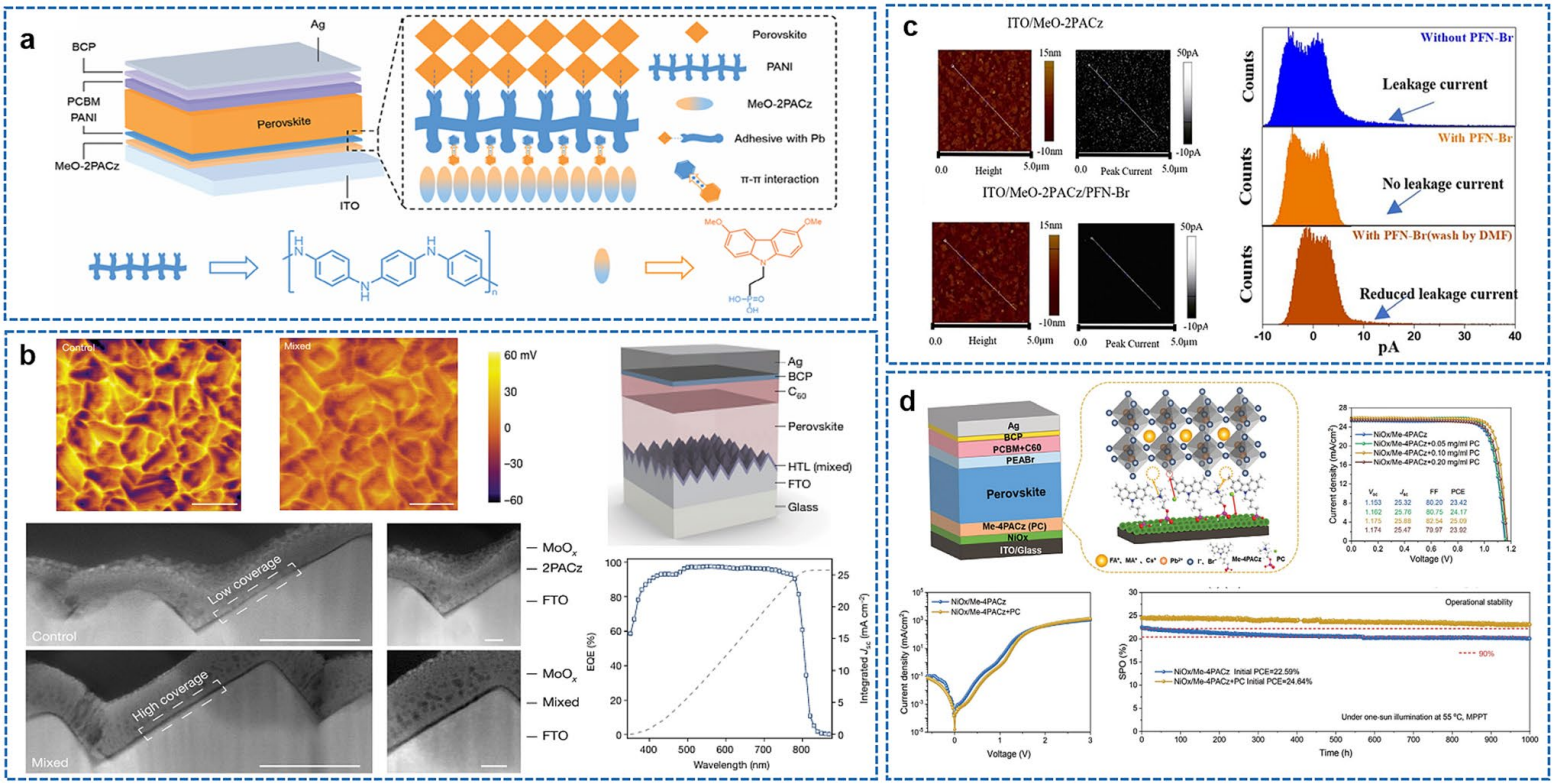
**a** Schematic diagram of the mechanism of conductive adhesive interaction [139]. Copyright 2024 Wiley–VCH; exclusive licensee American Association for the Advancement of Science. **b** KPFM images of control and mixed SAM-coated FTO substrates (left-top). Cross-sectional HAADF-STEM images of control and mixed SAMs sandwiched between MoO_*x*_ and FTO (left-bottom). Schematic illustration of the device architecture with textured FTO substrate (right-top). EQE and integrated *J*_SC_ curves of the mixed SAM device (right-bottom) [68]. Copyright 2023 Springer Nature Limited. **c** Conductive atomic force microscopy (C-AFM) images. Height and current map of ITO/MeO-2PACz and ITO/MeO-2PACz/PFN-Br (left). The histogram of the c-AFM map data (right) [134]. Copyright 2024 The Royal Society of Chemistry. **d** Schematic diagram of the inverted device structure and the interaction role of Co-SAM with NiO_x_ and perovskite films (top-left). Effect of Me-4PACz doped with different concentrations of PC on device performance (top-right). Dark *J-V* curves of PSCs with different substrates (bottom-left). SPO of the encapsulated PSCs measured at MPP under continuous 1-sun illumination (bottom-right) [135]. Copyright 2024 Wiley–VCH

Although SAMs have been extensively employed in PSCs and have yielded remarkable results, they continue to exhibit intrinsic limitations, as previously discussed. In recent years, significant research efforts have been devoted to further optimizing SAMs to enhance their functionality in PSCs (Fig. 12a). Park et al. [133] discovered that fluorinated anilines not only provide robust interfacial passivation but also minimize undesirable chemical reactions with the perovskite layer. Building on this insight, they employed a small molecule, 345Fan, to construct a 2D/3D heterojunction on the perovskite surface, effectively suppressing interfacial defects. As a result, the inverted PSCs achieved a certified quasi-steady-state PCE of 24.09%. Moreover, the encapsulated devices demonstrated outstanding thermal and environmental stability, maintaining 85% of their initial efficiency (T_85_) after 1560 h of continuous operation under one-sun illumination at 85 °C and 50% relative humidity (RH). In the same year, Park and co-workers [68] proposed a co-adsorption strategy aimed at disassembling high-order molecular clusters, wherein 3-mercaptopropionic acid (3-MPA) was employed to optimize the spatial distribution of phosphonic acid-based SAMs. This approach effectively suppressed interfacial recombination and optimized the interfacial electronic structure, leading to a certified PCE of 24.8%. Moreover, the encapsulated devices exhibited excellent durability, retaining 95% of their peak performance after over 1000 h of MPP tracking at 65 °C and 50% RH, as depicted in Fig. 12b. Liu et al. [134] introduced an amphiphilic conjugated polymer, PFN-Br, between the SAM and perovskite layers to minimize interfacial voids. This modification effectively enhanced surface coverage and reduced variability associated with buried interfacial gaps. As a result, the devices achieved a remarkable FF exceeding 0.84 and a PCE of over 22.5%. Moreover, across 50 individual cells, the devices exhibited an average FF of 0.81 with an exceptionally low standard deviation (< 0.01), demonstrating excellent reproducibility and consistent high performance, as presented in Fig. 12c. Similarly, Cao et al. [135] doped phosphorylcholine chloride (PC) into Me-4PACz. The phosphate and chloride (Cl^−^) ions in PC effectively suppressed surface defects on NiO_*x*_, while the quaternary ammonium cations and Cl^−^ ions filled organic cation and halide vacancies within the perovskite layer, enabling comprehensive defect passivation. As a result, the co-SAM–modified devices exhibited an impressive PCE of up to 25.09% and retained 93% of their initial efficiency, underscoring their superior performance and long-term stability, as illustrated in Fig. 12d. Zheng et al. [136] introduced (aminomethyl)phosphonic acid (AMP) into the precursor solution, where strong FA–AMP hydrogen bonding directed preferential FA crystallization, thereby balancing the overall crystallization process. AMP spontaneously migrated and anchored at the buried interface via covalent bonds between –PO_3_H_2_ and FTO, filling SAM voids, homogenizing the surface potential, and minimizing interfacial recombination losses. As a result, MA-free devices achieved a PCE of 25.35% and a *V*_OC_ of 1.17 V. Xu et al. [137] introduced a co-assembly material, 4-(aminomethyl)-N,N-diphenylaniline iodide (TPAI), to promote the orderly formation of SAMs. The –NH^3+^ groups coordinate with undercoordinated Pb^2+^ ions in CsPbI_3_, passivating buried-interface defects, optimizing energy alignment, and significantly enhancing hole extraction kinetics. The TPAI-treated CsPbI_3_ PSC achieved a PCE of 21.60% and retained 96.71% of its initial efficiency after 1400h of MPP tracking. Kim et al. [138] engineered CsPbI_3_ PSCs by functionalizing SAM-based hole-selective interfaces with MXene (Ti_3_C_2_T_*x*_) nanosheets to enhance conductivity, adjust anode WF, and improve surface properties. Integrating MXene facilitated efficient charge transport, reduced interfacial trap density, suppressed non-radiative recombination, and improved film quality, enabling a PCE of 23.25%. Systematic engineering of SAMs has proven pivotal in achieving record efficiencies in inverted PSCs, laying a foundation for their application in more complex and sophisticated architectures. Table 2 summarizes various SAM-modification strategies in PSCs, revealing that doping-based engineering strategies are particularly effective in improving the short-circuit current density (*J*_SC_) of the devices. This effect likely arises because additive molecules facilitate suppression of non-radiative recombination at the buried interface. Moreover, bilayer architectures generally yield higher FF values, which may be attributed to their ability to compensate for incomplete SAM coverage and imperfect molecular ordering during SAM deposition, while simultaneously guiding crystallization and nucleation behavior of perovskite films. Furthermore, post-treatment strategies tend to improve *V*_OC_, mainly via reducing interfacial defects and suppressing non-radiative recombination losses.

**Table 2:** Illustration of champion photovoltaic performance of inverted PSCs with different SAM modifications in recent years

SAMs	Methods	Additives	PCE (%)	*V*_*oc*_* (V)*	*J*_*sc*_* (*μA cm^−2^)	FF (%)	Refs.
MeO-2PACz	Additive/SAM/additive	Al_2_O_3_-NPs	26.31	1.190	26.10	84.70	[74]
Me-4PACz	Doped	BCPA	19.08	1.308	17.10	85.30	[55]
MeO-2PACz	Doped	F127	25.40	1.170	26.00	83.10	[140]
MeO-2PACz	Doped	FC-3283	25.70	1.190	25.91	83.50	[141]
2PACz	Doped	3-MPA	24.80	1.150	25.50	84.50	[68]
MeO-2PACz	Doped	Ti_3_C_2_T_*x*_	23.25	1.150	24.42	82.79	[138]
MeO-2PACz	Doped in PVK precursor	DMAcPA	25.69	1.179	25.50	85.49	[142]
MeO-4PACz	Doped in PVK precursor	AMP	25.35	1.170	25.77	84.02	[136]
N/A	Doped in PVK precursor	MeO-2PACz	12.92	1.140	15.03	75.33	[143]
N/A	Doped in PVK precursor	ATAA-DMAcPA	26.64	1.175	26.52	85.48	[144]
Me-4PACz	Bilayer	MPTMS	24.90	1.180	25.40	83.30	[38]
MeO-2PACz	Bilayer	PANI	25.59	1.220	24.90	83.90	[145]
MeO-2PACz	Bilayer	CB-PA	25.27	1.173	25.42	84.75	[146]
2PACz	Bilayer	PyCA-3F	25.12	1.170	25.22	86.00	[147]
2PACz	Bilayer	TPAI	21.60	1.260	20.74	82.95	[137]
MeO-2PACz	Bilayer	PFN-Br	22.50	1.090	24.40	84.00	[134]
2PACz	Bilayer	TATPA	26.08	1.185	26.27	83.84	[148]
MeO-2PACz	Bilayer	9-YT	24.82	1.170	25.56	82.92	[149]
Me-4PACz	Bilayer	FBA	25.58	1.186	25.78	83.70	[150]
Me-4PACz	Co-SAMs	PNPP	23.66	1.160	24.88	82.64	[57]
Me-4PACz	Co-SAMs	PC	25.09	1.175	25.88	82.54	[135]
DC-PA	Co-SAMs	IAHA	23.59	1.160	24.66	82.45	[151]
Me-4PACz	Co-SAMs	PLF	24.40	1.150	24.51	84.92	[152]
MeO-2PACz	Co-SAMs	Br-4PADBC	24.52	1.163	25.50	82.82	[142]
MeO-2PACz	Co-SAMs	TBA	23.05	1.178	23.44	83.44	[153]
MeO-2PACz	Post-treatment	MPA	22.91	1.250	22.18	82.60	[56]
Me-4PACz	Post-treatment	GPC	23.31	1.230	22.86	82.91	[75]
CNph	Post-treatment	POZ-BT-PY	24.45	1.169	25.31	82.63	[154]
Me-4PACz	Post-treatment	Br-EPA	25.16	1.20	25.10	83.54	[42]
MeO-4PACz	Post-treatment	EA	24.42	1.169	24.94	83.76	[155]

### Perovskite Tandem Solar Cells

Recent studies have extended SAM-HTLs to both all-perovskite and perovskite/silicon tandem cells, these TSCs deliver significantly higher PCEs than their single-junction counterparts. Nevertheless, their record efficiencies remain below the theoretical limit, and their operational stability continues to lag behind that of crystalline silicon solar cells. Among the key components, WBG perovskites have emerged as the most indispensable materials for tandem solar architectures. However, in WBG PSCs, microscale defects and interfacial non-uniformities at the buried interface frequently induce energy losses and inefficient charge extraction. In this context, SAMs have emerged as one of the most promising materials to address these interfacial challenges and enhance both performance and stability.

**Fig. 13:**
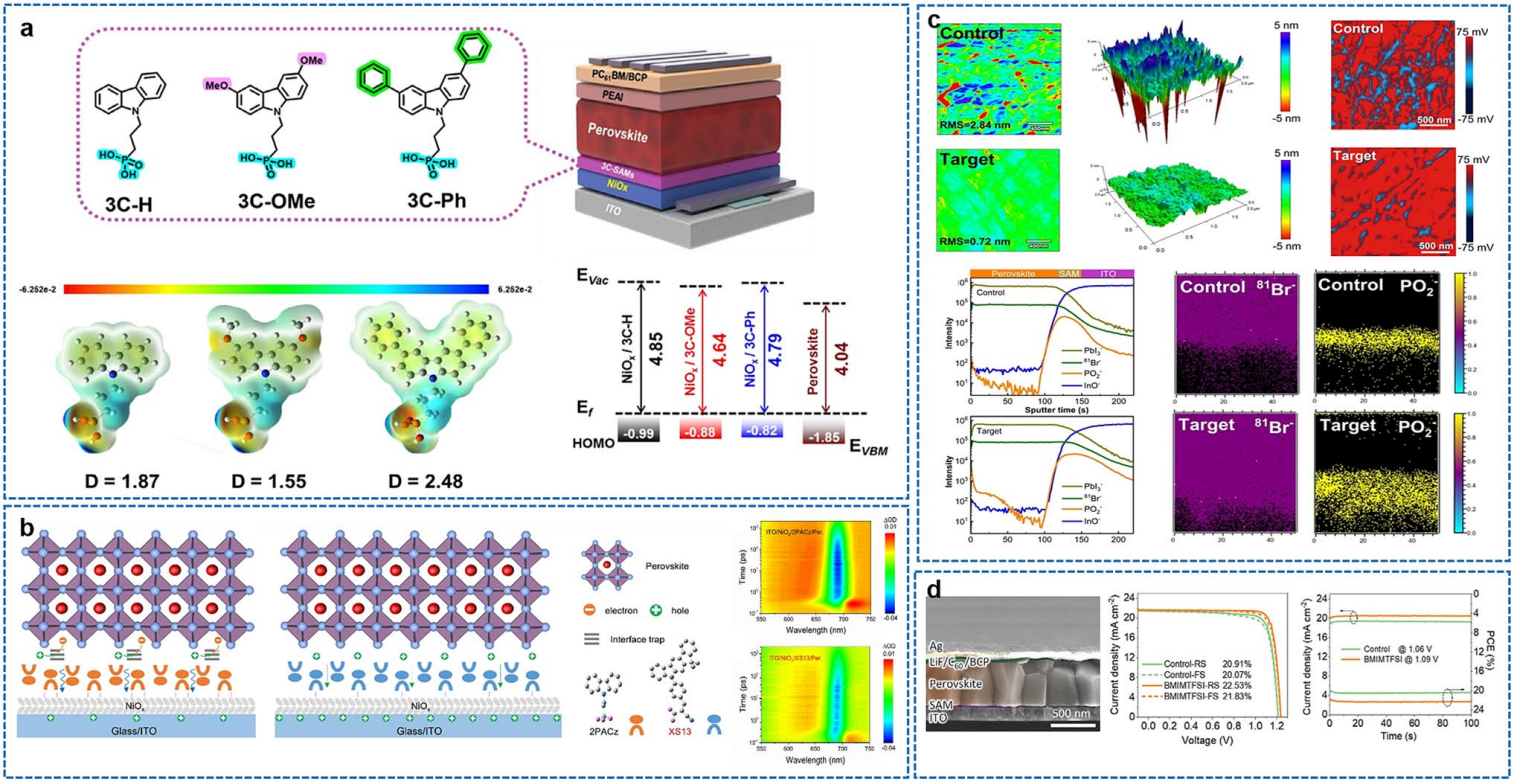
**a** Chemical structure of the studied 3C-SAMs materials and schematic of the demonstrated perovskite solar cell (top). Electrostatic surface potential (ESP) and molecular dipole moments (bottom-left). Energetic diagrams of 3C-SAMs materials and perovskite (bottom-right) [110]. Copyright 2025 Wiley–VCH. **b** Schematic illustration of hole transfer dynamics at interfaces (left). 2D color plot of TA spectra (right) [131]. Copyright 2024 Elsevier. **c** AFM and AFM-IR images of perovskite bottom interface (top). TOF–SIMS depth profiles of perovskite films (bottom-left) and negative ions two-dimensional-mapping of the samples (bottom-right) [140]. Copyright 2025 Elsevier. **d** Cross-sectional SEM image of the complete WBG PSC device (left). Champion *J-V* curves (middle). SPO for WBG PSCs (right) [141]. Copyright 2025 Wiley–VCH

Gnanasekaran [110] investigated a series of SAMs incorporating an n-propyl (3C) linker with various carbazole substituents, collectively referred to as 3C-Ph. When anchored on NiO_*x*_, 3C-Ph facilitated favorable energy-level alignment, enabling devices to deliver a high *V*_*OC*_ of 1.23 V and a maximum PCE of 21.59% under AM 1.5G illumination. Remarkably, under indoor lighting conditions, the 3C-Ph-based PSCs achieved a *J*_*SC*_ of 280.37 μA cm^−2^, *V*_*OC*_ of 1.09 V, and an FF of 81.43%, resulting in an impressive overall PCE of 41.77% (Fig. 13a). Guo et al. [131] introduced a novel SAM, termed XS13, to mitigate charge accumulation and suppress electron recombination in ~ 1.8 eV WBG PSCs. Devices incorporating XS13 exhibited significantly reduced light/electric-induced halide migration, resulting in significantly enhanced operational stability. To address the weak adsorption of SAMs onto ITO and the formation of surface vacancies caused by desorption of –OH groups induced by polar solvents, He and co-workers [140] introduced a dimethylacridine-based SAM directly into the perovskite precursor solution, as illustrated in Fig. 13c. This additive SAM displaced weakly bound molecules and occupied vacancies within the original HTL monolayer, thereby optimizing energy-level alignment, facilitating more enhanced hole extraction, and alleviating residual strain in the perovskite film. Feng’s team [141] incorporated an ionic liquid, 1-butyl-3-methyl-imidazolium bis(trifluoromethylsulfonyl)amide (BMIMTFSI), into the SAM solution, which reduced the film roughness, improved its wettability, and produced a more uniform surface potential distribution (Fig. 13d). The introduced ionic species also readily interacted with the perovskite lattice, effectively passivating undesirable cation and halide vacancies near the buried interface. By integrating non-transparent WBG PSCs with CuInGaSe_2_ solar cells, this strategy yielded a four-terminal tandem photodiode that achieved an impressive PCE of 28.34%. He et al. [142] sequentially deposited SAM materials onto NiO_x_, effectively filling interfacial voids and modifying the buried interface. This multilayer strategy produced a more compact and uniform interfacial contact compared to single-layer coating, eliminating the formation of small grains caused by random nucleation. Moreover, the refined interfacial structure facilitated energy-level alignment, enabling a champion WBG single-junction PSC to achieve an open-circuit voltage (*V*_OC_) of 1.33 V. Li and colleagues [143] adopted a similar dual-SAM blending strategy to enhance the adsorption energy of SAMs on ITO, thereby facilitating the formation of a dense and moisture-resistant HSL. This modification enhanced hole transport, enabling a WBG (1.68 eV) perovskite solar cell to achieve a PCE of 22.63% and an exceptionally high FF of 86.67%. Moreover, the encapsulated devices exhibited remarkable damp-heat stability (ISOS-D-3, 85% RH, 85 °C), maintaining 90% of their initial efficiency (T_90_) after 900h of continuous operation. For addressing similar interfacial challenges, Guo et al. [144] introduced 3-aminopropanoic acid (3-APA) into Me-4PACz, which improved the wettability of the perovskite precursor and optimized the buried interfacial morphology and homogeneity. This modification effectively suppressed non-radiative recombination and minimized interfacial energy losses, delivering a *V*_OC_ × FF equal to 84.5% of the Shockley–Queisser limit. As a result, the 1.67 eV WBG PSC achieved a PCE of 22.4%, accompanied by a *V*_OC_ of 1.255 V and an FF of 85.5%. Guo et al. [119] addressed the challenges of efficiency loss and instability in WBG PSCs caused by non-radiative recombination and degradation at the heterojunction interface by developing a novel SAM material, 4-(11H-benzo[a]carbazol-11-yl)butyl phosphonic acid (4-PhCz). This SAM demonstrated stronger adhesion to ITO and facilitated enhanced interfacial coupling with the perovskite layer. Consequently, the 1.67 eV WBG PSC achieved a high *V*_OC_ of 1.273 V, corresponding to a remarkably low voltage loss of 0.397 V relative to the bandgap, and achieved a PCE of 22.53%.

**Fig. 14:**
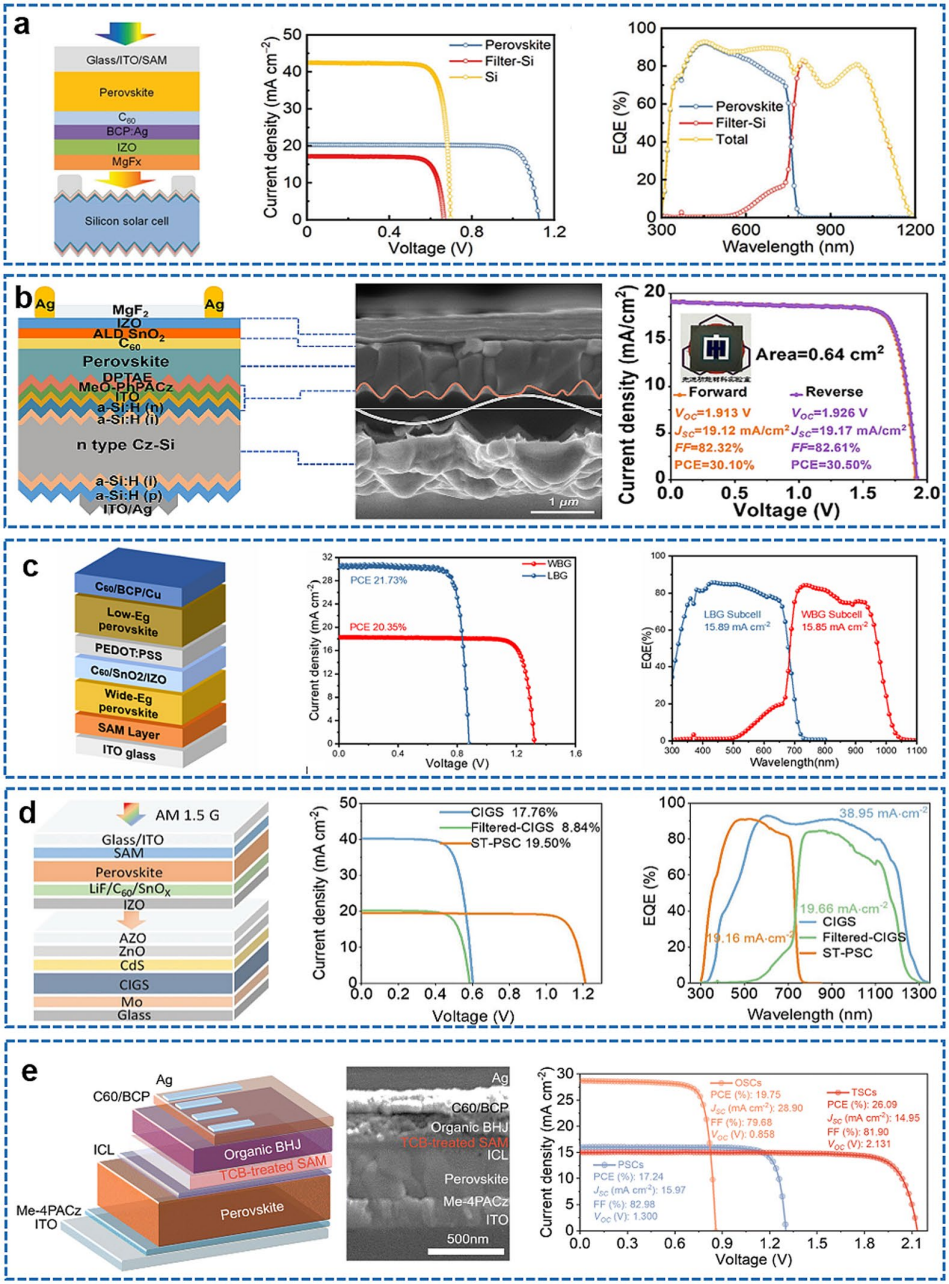
**a** Schematic of 4-terminal (4 T) perovskite/silicon tandem solar cells (left). *J-V* curves of the perovskite top cell and silicon bottom cell in the 4 T tandem configuration (middle). EQE spectra of the perovskite top cell and silicon bottom cell in the 4 T tandem configuration (right) [145]. Copyright 2023 Wiley–VCH. **b** Schematic illustration and cross-section SEM images of P/S-TSCs (left), and *J-V* curves with forward and reverse scanning directions of the champion DPTAE-based P/S-TSCs (right) [146]. Copyright 2025 Science Press and Dalian Institute of Chemical Physics, Chinese Academy of Sciences. **c** Device architecture of all-perovskite TSC (left). *J-V* curves (middle) and EQE curves of LBG and WBG subcells (right) [142]. Copyright 2025 American Chemical Society. **d** Schematic diagram of the 4 T-perovskite/CIGS tandem device configuration (left). *J-V* curves of the champion semitransparent WBG PSC and 4 T-perovskite/CIGS tandem solar cell (middle). EQE spectra of the CIGS, semitransparent WBG PSC device, and filtered CIGS (right) [141]. Copyright 2025 Wiley–VCH. **e** Schematic and cross-sectional SEM images of the device configuration of the TSC (left). *J–V* curves of champion OSCs, PSCs, and TSCs (right) [151]. Copyright 2025 The Royal Society of Chemistry

Notably, SAMs have been widely applied across various types of TSCs, where precise interfacial control is essential for achieving high-efficiency and long-term operational stability. Wu and co-workers [145] proposed an effective strategy to enhance SAM adhesion via selective removal of undesirable terminal hydroxyl groups and hydrolyzed residues from the ITO surface through hydrofluoric acid treatment followed by UV–ozone activation (Fig. 14a). This surface reconstruction significantly increased the surface activity and effective area of ITO, enabling high-density SAM adsorption. The resulting fluorinated surface simultaneously prevented direct ITO/perovskite contact and passivated the buried interface. Based on this approach, they fabricated PSCs with a PCE of 21.3%, a certified semitransparent PSC achieving 19.0% efficiency with a record FF of 84.1%, and a four-terminal perovskite/silicon tandem device delivering an efficiency of 28.4%. Li et al. [146] introduced a new V-shaped molecule, 4,4′-(perfluorocyclopent-1-ene-1,2-diyl)bis(N,N-bis(4-methoxyphenyl)aniline) (DPTAE), at the buried perovskite interface (Fig. 14b). Notably, DPTAE disrupted excessive SAM aggregation and formed an amorphous hole-selective layer, owing to its non-planar, sterically hindered structure. This favorable interfacial configuration enhanced charge extraction and minimized interfacial defect states. Consequently, the optimized 1.68 eV WBG PSC achieved an impressive PCE of 22.33%, while the DPTAE-based two-terminal monolithic perovskite/silicon TSCs delivered a remarkable efficiency of 30.50%. Similarly, Yan et al. [121] reported a chiral helical SAM, aza-helicene phosphonic acid (A7HPA), whose self-assembly is governed by the extended non-planar-conjugated helical backbone. The enhanced torsion and molecular-level helicity produced highly stable monolayers, exhibiting strong thermal, photochemical, and electrochemical robustness. Incorporation of A7HPA endowed perovskite–Si tandems with outstanding long-term stability under damp heat and light stress, achieving a PCE of 33.06%. He et al. [142] also modified NiO_*x*_ via SAMs, and on this basis, fabricated all-perovskite TSCs (ATSCs) with a PCE of 27.03% (Fig. 14c). Li’s group [123] developed a bidentate-anchored, superwetting aromatic SAM, ((9H-carbazole-3,6-diyl)bis(4,1-phenylene))bis(phosphonic acid) (2PhPA-CzH). The incorporation of dual phosphonic anchoring groups provides enhanced adsorption and efficient hole extraction/transport. However, the substituted carbazole unit imparts ultrahigh wetting, enabling the formation of high-quality perovskite films with minimal energetic mismatch. Collectively, these interfacial advantages drive the device to an impressive PCE of 32.19% at an active area of 1 cm^2^, while sustaining excellent photostability under prolonged operation. Zhu and colleagues [147] developed a custom-designed SAM tailored for tin (Sn)-based PSCs, extending its application to efficient Sn–Pb PSCs and all-perovskite tandem architectures. Oligoether side chains integrated into the SAM accelerated hole extraction, modulated perovskite crystallization, and effectively passivated surface defects in Sn–Pb alloyed perovskites. Consequently, the engineered LBG PSC achieved a champion PCE of 23.54%, while the corresponding monolithic all-perovskite tandem reached an impressive efficiency of 28.61%, featuring a *V*_OC_ of 2.155 V and an excellent operational stability. A four-terminal tandem configuration further delivered 28.22% efficiency. Jiao et al. [148] introduced the multifunctional molecule, (Z)-4-fluoro-N′-hydroxybenzimidamide (4F-HBM) at the SAM and WBG perovskite interface to regulate crystal growth and enhance hole extraction. Hydrogen bonding between the F atom of 4F-HBM and the SAM, coupled with coordination between 4F-HBM and Pb^2+^, effectively minimized interfacial defect density and curtailed non-radiative recombination. Leveraging this interfacial engineering, a four-terminal all-perovskite tandem device, integrating the WBG subcell with a 1.25 eV LBG PSC, delivered a PCE of 28.71%. Collectively, these results validate the compatibility of SAM-based HTLs with tandem architectures, offering a promising avenue toward further efficiency improvements. Feng et al. [141] combined WBG PSCs with CuInGaSe_2_ solar cells to construct four-terminal tandem photovoltaic devices, which achieved a champion PCE of 28.34% (Fig. 14d). An’s group [149] proposed an SAM/MoO_3_/SAM sandwich HTL that effectively converted the intrinsically n-type MoO_3_ surface into p-type, thereby facilitating efficient hole extraction, promoting more balanced carrier transport, and significantly suppressing non-radiative recombination at the interconnection layer. The resulting perovskite/organic TSCs achieved a PCE of 26.05% with a certified *V*_OC_ of 2.216 V. Chen and co-workers [150] passivated defects in nickel oxide using benzylphosphonic acid, thereby suppressing interfacial recombination, which resulted in the increase in the voltage of the 1.79-eV bandgap perovskite subcell to 1.26 V. Based on this strategy, they further achieved a certified efficiency of 22.95% for perovskite/organic TSCs. Sun and colleagues [151] induced highly ordered stacking of SAMs by leveraging the interaction between the *π*-conjugated backbone of SAMs and volatile solid additives with opposing electrostatic potentials, as illustrated in Fig. 14e. Consequently, a PCE of 26.09% was achieved in perovskite-organic TSCs. Yoon’s team [152] found that nicotinic hydrazide (NH) could form an energetically favorable complex with phosphonic acid groups, thereby eliminating aggregated 2PACz and generating a uniform and aggregation-free SAM layer. As a result, the fabricated TSC achieved a PCE of 19.89% with an active area of 16.41 cm^2^.

### Large-Area Perovskite Solar Modules

Although single-junction and tandem PSCs have demonstrated outstanding performance efficiency, the upscaling of these SAM-based PSCs into large-area modules remains challenging. Issues such as poor wetting of the perovskite precursor on SAM surfaces, the difficulty of achieving highly ordered monolayers for efficient hole transport, and weak interfacial adhesion between SAMs and the perovskite collectively limit their compatibility with large-area device fabrication. Moreover, efficiency losses in perovskite solar modules (PSMs) primarily stem from increased series resistance, interconnection losses associated with P1/P2/P3 laser patterning, and non-uniform perovskite film formation across large-area substrates. Interfacial deficiencies typically manifest as inferior charge transport, increased carrier recombination, and a decline in FF. Incorporating SAMs provides a means to optimize interfacial energy-level alignment and facilitate charge extraction across large areas, thereby alleviating resistive losses. Moreover, the ordered molecular arrangement and interfacial interactions of SAMs help regulate large-area perovskite crystallization and improve film uniformity, thus suppressing defect accumulation and non-radiative recombination in PSMs [153]. Recent studies have begun to explore SAM-based HTLs as a strategy for enhancing the performance of large-area devices.

**Fig. 15:**
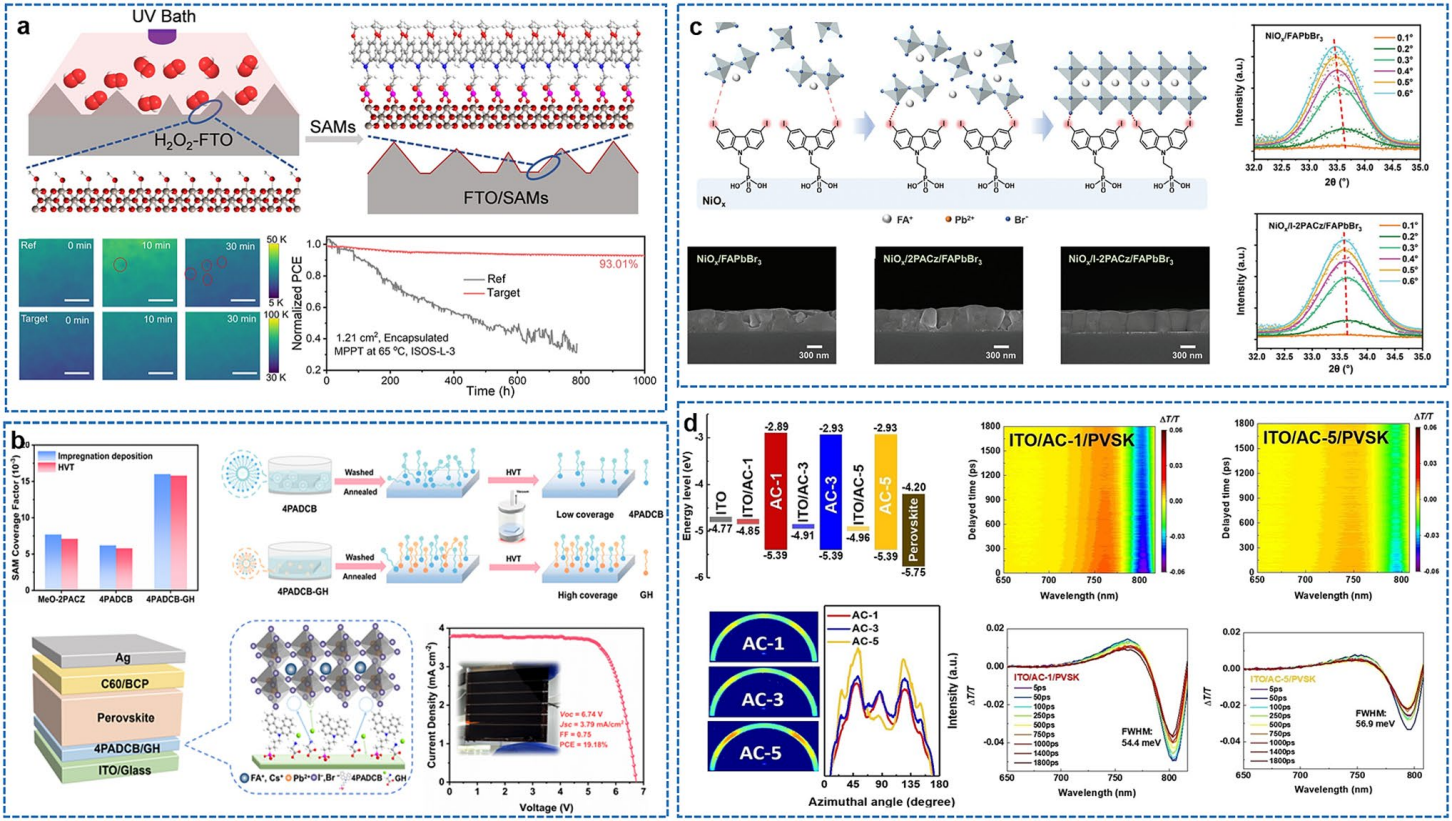
**a** Schematic of seeding ─OH groups via H_2_O_2_/UV treatment (top). Micro-PL mapping of the films under 1-sun 532 nm laser illumination from the glass side (bottom-left). Operational stability of the large-area devices under simulated AM 1.5 illumination at 65 °C (bottom-right) [154]. Copyright 2025 The Royal Society of Chemistry. **b** Coverage factor of SAMs deposited on ITO and after HVT and schematic diagram for mixed self-assembled monolayers with impregnation method fabrication (top). Schematic diagram of the modified inverted device structure and the interaction role of 4PADCB-GH with perovskite films (bottom-left). Champion *J-V* curves of the 5 cm × 5 cm mini-module with a 10 cm^2^ mask measured under simulated AM 1.5G 1 sun illumination (bottom-right) [155]. Copyright 2025 American Chemical Society. **c** Schematic diagram of halogen-halogen bond formation between I-2PACz and FAPbBr_3_ at the buried interface and its impact on the perovskite crystallization (left-top). Cross-sectional SEM images (left-bottom) and depth-dependent GIXRD patterns of FAPbBr_3_ films on different SAMs (right) [156]. Copyright 2024 Wiley–VCH. **d** Energy-level diagram of different compositions in PSCs (left-top). 2D GIWAXS patterns and azimuth integral with an incident angle of 0.2° (left-bottom). Pseudo-color plots of the picosecond transient absorption (ps-TA) spectra for perovskites atop AC-series SAM-coated ITO (right-top). ps-TA spectra of perovskites atop AC-series SAM-coated ITO under front face excitation (right-bottom) [157]. Copyright 2023 Wiley–VCH

**Fig. 16:**
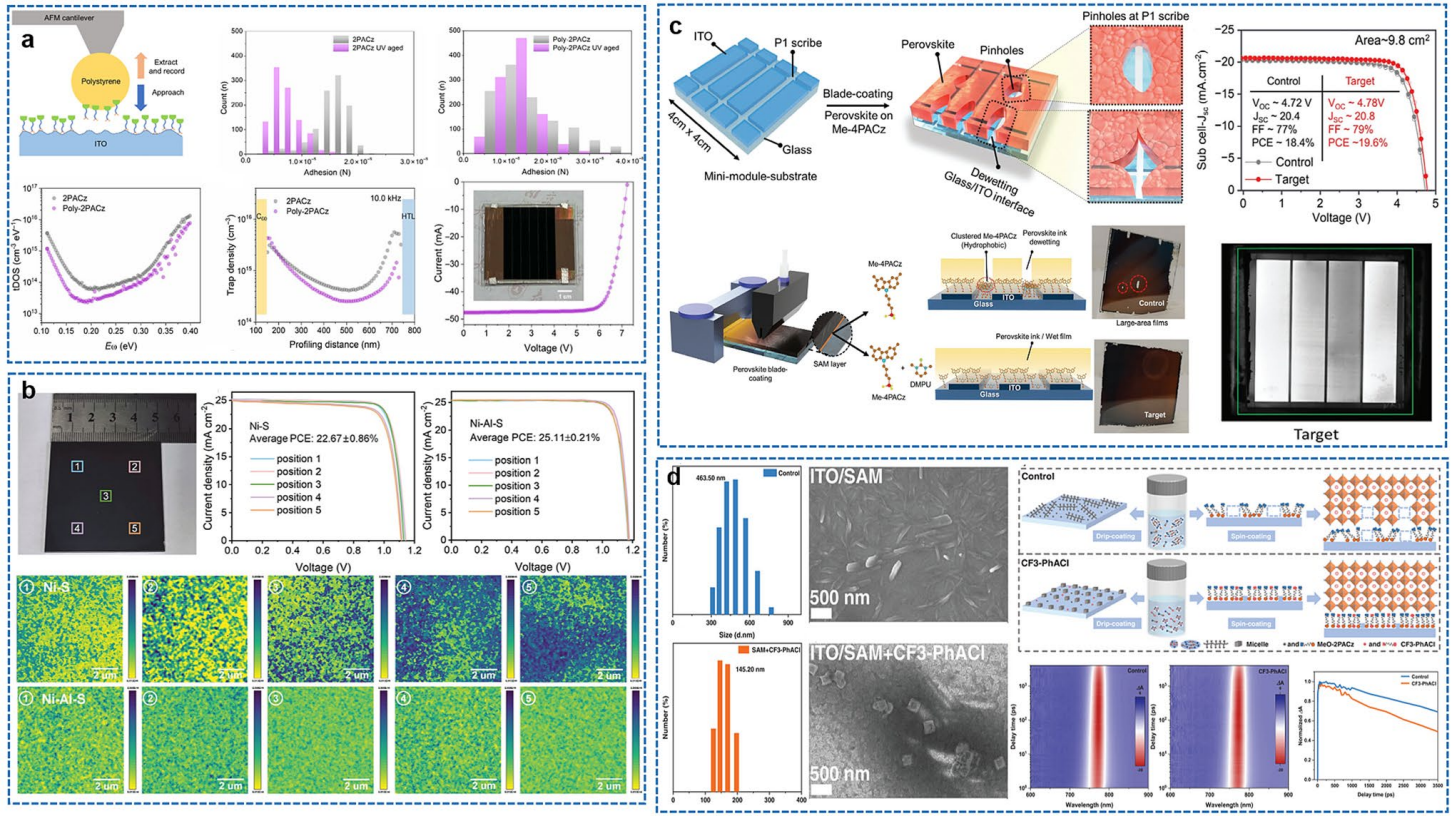
**a** Schematic illustration of AFM adhesion force measurement on HTLs (top-left), and adhesion force distribution on ITO substrates (top-right). tDOS spectra (bottom-left) and DLCP trap profiling (10.0 kHz) of the PSCs. (bottom-middle) *I-V* curve of champion perovskite mini-module (12.25 cm^2^ aperture area) based on Poly-2PACz (bottom-right) [159]. Copyright 2025 The Authors, some rights reserved; exclusive licensee American Association for the Advancement of Science. **b** Optical image of large-area perovskite films and schematic diagram of selecting five different positions (top-left). *J-V* curves of PSCs prepared at five different positions of large-area perovskite films (top-right). TRPL mapping images at the buried interface (bottom) [161]. Copyright 2024 Wiley–VCH. **c** Schematic highlighting the module design and coating challenges on Me-4PACz at P1 scribe areas, illustrating pinholes (left-top). Blade-coating of perovskite ink on large-area substrates (left-bottom). *J-V* of control and target devices with champion mini-module performances (right-top). EL imaging of the target devices (right-bottom) [160]. Copyright 2024 Wiley–VCH. **d** Particle size distribution in SAM solutions determined through DLS characterization and top-view SEM pictures of SAM micelles on ITO substrates dried at room temperature without the spin-coating and annealing treatment (left). Schematic illustration for the evolution of SAM micelles on ITO substrates (right-top). The pseudo-color TA spectra for the buried interface of modified perovskite films and the normalized charge-carrier kinetics of the corresponding GSB peaks (right-bottom) [163]. Copyright 2025 Wiley–VCH

As depicted in Fig. 15a, Fu et al. [154] found that SAMs preferentially formed strong –P–O–Sn bonds with hydroxyl groups (–OH) on metal oxides, enhancing stable adsorption. They used H_2_O_2_/UV treatment to seed –OH groups on the substrate, which enhanced both the uniformity and packing density of SAM deposition, yielding a module with an active area of 13.8 cm^2^ and a PCE of 21.77%. As presented in Fig. 15b, Hu and co-workers [155] employed a vapor-phase deposition approach to introduce SAMs as HTLs. To address the migration of physisorbed SAM molecules that had not yet formed covalent bonds during the solid–gas reaction, they developed an impregnation method for mixed SAMs composed of 4-(7H-dibenzo[c,g]carbazol-7-yl)butyl phosphonic acid (4PADCB) and glycine hydrochloride. This strategy minimized SAM molecular aggregation and significantly strengthened their anchoring on the substrate, enabling high SAM coverage even under high-temperature and low-pressure vapor-phase reaction conditions. Consequently, devices fabricated by this method delivered PCEs of 22.15% for small-area cells (0.16 cm^2^) and 19.18% for a 5 cm × 5 cm module, demonstrating the method’s promising potential for scalable fabrication. As illustrated in Fig. 15c, Zhu et al. [156] investigated the role of SAMs in bromide-based PSCs, incorporating (2-(3,6-diiodo-9H-carbazol-9-yl)ethyl)phosphonic acid (I-2PACz) into FAPbBr_3_-based PSC architectures. I-2PACz alleviated intrinsic tensile strain in FAPbBr3, promoted oriented crystallization, and minimized defects through stabilizing halogen–halogen interactions. Moreover, it improved energy-level alignment at the hole-selective interface, facilitating more efficient hole extraction. Consequently, the devices achieved a PCE of 11.14%. Moreover, semitransparent devices and modules delivered impressive efficiencies of 8.19% and 6.23%, with average visible transmittances of 41.98% and 38.99%, respectively. The encapsulated cells maintained 93% of their initial PCE after 1000h of MPP tracking. Hung et al. [157] presented a novel class of carboxylic acid-functionalized porphyrin derivatives, introducing—for the first time—a strategy utilizing the extended π-conjugated porphyrin framework as a backbone for interfacing the ITO electrode and the perovskite active layer in inverted PSCs (Fig. 15d). Electron-rich porphyrin moieties facilitated efficient hole transfer, promoted the assembly of robust SAMs, passivated perovskite surface defects, and functioned as effective conduits for hole transport. This molecular design led to a significant enhancement in PSC performance, demonstrating the strong potential of porphyrin-based SAM architectures. Zhang et al. [124] developed a sterically hindered SAM, 4PA-spiro, featuring an orthogonal spiro[acridine-9,9′-fluorene] backbone and phosphonic acid anchoring groups. Its twisted molecular configuration suppresses intermolecular aggregation and promotes uniform substrate anchoring. Its suitable HOMO energy level facilitates efficient hole extraction while simultaneously reducing non-radiative recombination pathways. Large-area PSCs and modules achieved PCEs of 24.11% (1.0 cm^2^) and 21.89% (29.0 cm^2^), respectively. Tong et al. [125] reported an asymmetric π-extended SAM, (4-(9Hdibenzo[a,c]carbazol-9-yl)butyl)phosphonic acid (A-4PADCB), which exhibited a significant orientation angle away from the surface normal, homogenizing the distribution of contact potentials. This enhanced orientation improved SAM/perovskite interfacial quality and guided the growth of perovskite films with low defect density and slight tensile strain. Therefore, integration of A-4PADCB into large-area flexible PSMs with an aperture area of 20.84 cm^2^ achieved impressive PCEs of up to 20.64%. Tian et al. [158] reported that both the imino (N–H) and methoxy (CH_3_O–) groups in dimethyl suberimidate dihydrochloride (DMSCl_2_) could form bonds between the perovskite precursor and the Me-4PACz-SAM. By doping this additive into the perovskite precursor, large-area printed PSC modules (93.10 cm^2^) achieved a significant enhancement in PCE from 16.62% to 20.37%. Moreover, the devices exhibited excellent operational stability, retaining 93.3% of their initial efficiency after 1000 h of continuous MPP tracking. Zhang and co-workers [54] demonstrated that co-assembled molecules functionalized with a diamide terminal group established supramolecular interactions with commonly used carbazole-based SAMs. This cooperative assembly modulated the structural ordering of the SAMs while simultaneously improving chemical bonding with the perovskite Pb–I framework, thereby enhancing long-term device stability. Liu et al. [159] emphasized that in inverted PSCs, where incident light must traverse the HTL before reaching the active layer, UV radiation poses a severe challenge to device stability, necessitating the development of more robust HTL materials. To address this challenge, they designed and synthesized Poly-2PACz, a polymer exhibiting both strong substrate adhesion and distinct UV resistance. PSCs incorporating Poly-2PACz achieved a high power conversion efficiency of 26.0%, while blade-coated mini-modules demonstrated an aperture efficiency of 22.2% with excellent uniformity (Fig. 16a).

Liu’s team [161] employed a NiO_*x*_/mesoporous Al_2_O_3_ sponge scaffold to anchor the SAM at the buried interface, integrating this with dip-coating. This approach effectively passivated large-area buried interfacial defects and improved wetting, yielding mini-modules (10–200 cm^2^) with a champion PCE of 22.66% under ambient air conditions. Similarly, Zhao et al. [162] used an ultrasonic spray-coating method to deposit high-quality MAPbI_3_ perovskite films on SAMs. They found that the presence of SAM regulated perovskite crystallization, improving film quality and carrier extraction, ultimately enabling large-area modules (100 mm × 100 mm) with a PCE of 18.83%. For perovskite modules, Pininti et al. [160] demonstrated that perovskite ink around the P1 scribing region on glass surface can disrupt film uniformity and reproducibility. By integrating 1,3-dimethyl-3,4,5,6-tetrahydro-2(1H)-pyrimidinone (DMPU) into the SAM, they achieved enhanced precursor wetting, enabling the fabrication of highly reproducible mini-modules (9.8 cm^2^) with a PCE of 20% (Fig. 16c). Zhang and colleagues [163] observed that conventional SAM solutions tend to form poorly dispersed fibrous micelles, as depicted in Fig. 16d. By introducing CF3-PhACl, they achieved the formation of uniform, highly ordered, and high-coverage SAM films, yielding inverted PSCs (0.09 cm^2^) and modules (14.40 cm^2^ aperture area) with PCEs of 26.58% and 22.95%, respectively. Shi’s group [164] demonstrated that the co-adsorbent 6-aminohexane-1-sulfonic acid (SA) can occupy residual surface regions without disrupting Me-4PACz assembly, thereby forming a denser hole-selective layer. The resulting SA/Me-4PACz mixed SAM effectively suppressed non-radiative recombination at the buried interface, optimized energy alignment, and modulated the crystallization behavior of WBG perovskites. As a result, they fabricated 2-terminal all-perovskite TSCs, achieving a PCE of 28.94% for a 0.087 cm^2^ device and 23.92% for a mini-module with an aperture area of 11.3 cm^2^. Despite these advances, translating the interfacial benefits of SAMs from laboratory-scale devices to large-area modules requires further innovations in deposition methods and stability enhancement.

## Future Perspectives of SAMs

Despite the significant advances achieved with SAMs in inverted PSCs, several critical challenges must be addressed before their full potential can be harnessed in both laboratory-scale devices and large-scale industrial applications. These problems stem from the intrinsic molecular characteristics of SAMs, the intricate nature of buried interfaces, and the stringent requirements for long-term operational stability and scalable large-area fabrication.

At present, the key challenges of SAMs involve achieving uniform and robust surface coverage on practical substrates, while simultaneously ensuring strong interfacial binding strength and long-term chemical stability. In most cases, SAM-based interlayers are not perfectly defined monolayers, but rather mixed ensembles composed of covalently anchored molecules and weakly adsorbed species. The limited intrinsic stability of these mixed ensembles has emerged as a critical bottleneck, constraining further advances in device performance. Undeniably, a lot more systematic explorations are further demanded to develop more stable SAM molecules, which requires an in-depth understanding of the complete assembly configuration of SAMs as well as the dynamic process of molecular assembly [165], and it will be pursued in the future. Moreover, owing to the limited sensitivity of available characterization techniques for probing SAMs, the current understanding of the stacked microstructure within SAMs remains incomplete. In particular, most investigations into SAMs commence only after the molecular layer has been established, leaving the dynamic mechanisms underlying self-assembly process largely unexplored. However, comprehensive understanding of the dynamic self-assembly behavior of SAMs is crucial for the systematic screening of SAM species, deposition processes, and other related factors. Furthermore, this issue is also intrinsically associated with the future realization of large-scale industrial fabrication of devices. From the standpoint of device physics, the fundamental mechanisms governing charge transport across SAM-modified interfaces remain insufficiently understood. The ultrathin nature of SAMs raises fundamental questions regarding charge tunneling, dipole-induced band bending, and the role of molecular packing density in governing hole extraction. Simplified models that treat SAMs merely as passive dipole layers risk overlooking the complex interplay among molecular orientation, interfacial electronic states, and substrate-induced polarization effects. A comprehensive understanding of charge transport across SAM interfaces, and of how molecular-scale variations translate into macroscopic device parameters, is critical for rational interface design. Moreover, intrinsic trade-offs in molecular design impose additional constraints. For instance, tailoring SAMs to achieve deeper HOMO levels, favorable for maximizing open-circuit voltage, often compromises other critical attributes, such as surface wettability or interfacial binding strength. Conversely, denser molecular packing and stronger anchoring stability may compromise interfacial energetics or film formation of the perovskite absorber. These competing requirements make it difficult for a single-component SAM to simultaneously satisfy all performance and stability criteria.

Although SAMs have been extensively employed as HTLs in PSCs, developing efficient SAM-based ETLs continues to pose significant challenges. Common fullerene-based SAMs are hindered by poor solubility as well as complex synthesis and purification processes, whereas benzene-based SAMs continue to encounter challenges in processability, deposition compatibility, and precise control over molecular orientation and packing density. Moreover, although OSCs share structural similarities with PSCs, the use of SAM-based interlayers remains relatively rare, largely due to stability concerns such as SAM degradation and undesirable interfacial reactions with photoactive layers.

In summary, while SAMs have established themselves as powerful interfacial modifiers for high-performance inverted perovskite solar cells, addressing persistent challenges in achieving uniform coverage, ensuring long-term stability, deepening the understanding of charge transport, and scalable fabrication remain crucial. Continued advances in molecular design, co-SAM strategies, and interfacial engineering are expected to unlock the next stage of development of SAM-based PSCs.
